# HMGA1-TRIP13 axis promotes stemness and epithelial mesenchymal transition of perihilar cholangiocarcinoma in a positive feedback loop dependent on c-Myc

**DOI:** 10.1186/s13046-021-01890-1

**Published:** 2021-03-01

**Authors:** Zhipeng Li, Jialiang Liu, Tianli Chen, Rongqi Sun, Zengli Liu, Bo Qiu, Yunfei Xu, Zongli Zhang

**Affiliations:** 1 Department of General Surgery, Qilu Hospital, Cheeloo College of Medicine, Shandong University, 107 Wenhuaxi Road, Jinan, 250012 Shandong China; 2 Department of General Surgery, Shandong Second Provincial General Hospital, Shandong Provincial ENT Hospital, Jinan, China

**Keywords:** Perihilar cholangiocarcinoma, HMGA1;TRIP13, Prognostic biomarker, Epithelial-mesenchymal-transition, Stemness

## Abstract

**Background:**

Cholangiocarcinoma is a highly malignant cancer with very dismal prognosis. Perihilar cholangiocarcinoma(pCCA) accounts for more than 50% of all cholangiocarcinoma and is well-characterized for its low rate of radical resection. Effects of radiotherapy and chemotherapy of pCCA are very limited.

**Methods:**

Here we screened potential biomarkers of pCCA with transcriptome sequencing and evaluated the prognostic significance of HMGA1 in a large cohort pCCA consisting of 106 patients. With bioinformatics and in vitro*/vivo* experiments, we showed that HMGA1 induced tumor cell stemness and epithelial-mesenchymal-transition (EMT), and thus facilitated proliferation, migration and invasion by promoting *TRIP13* transcription. Moreover, TRIP13 was also an unfavorable prognostic biomarker of pCCA, and double high expression of HMGA1/TRIP13 could predict prognosis more sensitively. TRIP13 promoted pCCA progression by suppressing *FBXW7* transcription and stabilizing c-Myc. c-Myc in turn induced the transcription and expression of both *HMGA1* and *TRIP13*, indicating that HMGA-TRIP13 axis facilitated pCCA stemness and EMT in a positive feedback pathway.

**Conclusions:**

HMGA1 and TRIP13 were unfavorable prognostic biomarkers of pCCA. HMGA1 enhanced pCCA proliferation, migration, invasion, stemness and EMT, by inducing *TRIP13* expression, suppressing *FBXW7* expression and stabilizing c-Myc. Moreover, c-Myc can induce the transcription of *HMGA1* and *TRIP13*, suggesting that HMGA-TRIP13 axis promoted EMT and stemness in a positive feedback pathway dependent on c-Myc.

**Supplementary Information:**

The online version contains supplementary material available at 10.1186/s13046-021-01890-1.

## Background

Cholangiocarcinoma (CCA) is a highly malignant cancer originating from the bile ducts [1, 2]. According to the anatomical location and treatment options for CCA, CCA can be further divided into three subtypes, i.e., intrahepatic (iCCA), perihilar (pCCA), and distal CCA (dCCA) [3]. pCCA, accounting for more than 50% of all CCA cases [4], arises from the second-order intrahepatic bile ducts to the converging point of the cystic duct and the common bile duct. The preferred treatment for pCCA is surgical resection; however, radical resection of pCCA is extremely difficult because of the rapid progression and anatomical complexity of the hilus hepatis. Moreover, most patients present with jaundice, indicating that the stage is too late for radical resection. In general, for patients with advanced or unresectable CCA, the median overall survival is less than 1 year [5]. Even after radical resection, the 5-year overall survival rate is less than 30% [6]. The effects of radiotherapy and chemotherapy of pCCA are very limited, and studies on pCCA are far behind those of other more common tumors such as lung cancer and colon cancer in this era of precision treatment [7]. Several reasons can be attributed to, including (i) the prevalence of pCCA is relatively low, making a large cohort difficult to establish; (ii) pCCA specimens are hard to obtain because of its special anatomical location, resulting in rare reports on pCCA high-throughput experiments; (iii) most patients lose surgical opportunity and the survival times are so short to perform any experimental treatment. Thus, further studies of the pathogenesis and therapeutic options of pCCA are necessary.

Targeted therapy and precision treatments are based on effective biomarkers and an in-depth understanding of tumor progression. However, this is extremely hard as to pCCA because of the difficulty of specimen obtainment and establishment of large cohort. Even in the low proportion of patients who underwent resection, rate of radical resection is very low(< 30%) [8]. This increases the heterogeneity of surgical treatment and results in difficulties in collecting a homogeneous cohort to verify biomarkers or treatments. From our experience, we obtained a validation cohort of patients with pCCA who underwent radical resection (*n* = 106 patients), and confirmed several potential biomarkers of pCCA [9, 10]. However, more prognostic biomarkers of pCCA are needed to predict the post-operational risk and guide the individual treatment for patients with pCCA.

High mobility group A1 (HMGA1) protein is a small nuclear protein that acts as a structural transcription factor [11]. Under normal conditions, high expression of HMGA1 can occur during embryogenesis and in normal embryonic stem cells and adult stem cells [12, 13] In mature differentiated tissues, HMGA1 is barely detected; however, some ectopic events in cancer, such as oncogenic transcription factors, epigenetic changes, and chromosomal translocation events, can induce abnormal up-regulation of HMGA1 [14]. Ectopic expression of HMGA1 has been widely reported in different tumors, and overexpression of HMGA1 is associated with progression or poor prognosis in several types of cancers, including pancreatic adenocarcinoma [15], lung cancer [16], breast cancer [17], colon cancer [14], gastric cancer [18], and hepatocellular carcinoma [19]. Moreover, HMGA1 has been shown to activate a variety of genes involved in tumorigenesis, tumor proliferation, migration, invasion, and epithelial-mesenchymal transition [12]. In iCCA, HMGA1 was expressed and can enhance the tumorigenicity [20, 21]. However, the clinical significance of HMGA1 in pCCA has not been elucidated.

Accurate chromosome segregation is essential to avoid chromosomal aneuploidy, which is a common feature of human malignancies, accounting for approximately 90% of human solid tumors and more than 50% of hematopoietic tumors [22]. As a key modulator of chromosome segregation, thyroid hormone receptor interactor 13 (TRIP13) is expressed in various adult tissues and plays key roles in inactivation of the mitotic checkpoint complex (MCC) [23]. TRIP13 is an onco-protein in several types of cancers and is abnormally expressed in various human tumors, including head and neck cancer, hepatocellular carcinoma, colorectal cancer, breast cancer, etc. [24]. As a central protein in MCC inactivation, TRIP13 promotes tumor progression mainly by altering the conformation of the terminal macromolecule [25]. Overexpression of TRIP13 in nonmalignant cells can increase the tumorigenicity of the cells; however, the role of TRIP13 in pCCA are still unclear.

Accordingly, in this study, we evaluated the expression and clinical significance of HMGA1 in a large cohort of patients with pCCA and examined the oncogenic functions of HMGA1. With in vitro and in vivo experiments and bioinformatics, we identified TRIP13 as the key protein in HMGA1-induced stemness, EMT and metastasis of pCCA. In addition, we investigated the underlying mechanism of how HMGA1-TRIP13 axis promoted the progression of pCCA, and demonstrated that HMGA1-TRIP13 axis had a positive feedback loop dependent on c-Myc involvement.

## Materials and methods

### Retrospective cohorts and follow-up

The primary cohort comprised 325 patients who were diagnosed with pCCA at Qilu Hospital of Shandong University and underwent surgical resection from 2013 to 2018. A validation cohort consisting of 106 patients with pCCA was further selected from the primary cohort according to the following inclusion criteria: available pCCA tumor tissues for further study; survival time more than 1 month; no history of chemotherapy or radiotherapy; and no history of other malignancies. The basic information for the primary and validation cohorts is described in Supplementary Table 1. All patient materials were obtained after obtaining informed consent, and the study was approved by the Clinical Research Ethics Committees of Shandong University.

### Cells and reagents

HIBEpic human biliary epithelial cells, pCCA cell lines (QBC939 and FRH0201), RBE iCCA cells, and gallbladder cancer cell lines (GBC-SD, NOZ, and SGC-996) were purchased from the Chinese Academy of Sciences Cell Bank (Shanghai, China). HCCC-9810 iCCA cells were purchased from American Type Culture Collection (Manassas, VA, USA). All cell lines were cultured according to the instructions recommended by the cell banks.

Anti-HMGA1 antibodies (cat. no. ab129153), anti-TRIP13 antibodies (cat. no. ab204331), anti-Fbxw7 antibodies (cat. no. Ab109617), anti-laminB1 antibodies (cat. no. ab16048) and anti- OCT4 antibodies (cat. no. Ab19857) were purchased from Abcam (Cambridge, UK). Anti-c-Myc antibodies (cat. no. YT0991), anti-TCF4/TCF12 antibodies (cat. no.YT4580) and anti-GAPDH antibodies (cat. no. YM3215) were purchased from ImmunoWay Biotechnology (Plano, TX, USA). Anti-EMT antibodies kit (cat. no. 9782 T) and anti-CD44 antibodies (cat. no. 3570S) were purchased from Cell Signaling Technology. All other agents were from Sigma-Aldrich (St. Louis, MO, USA).

### Tissue microarray (TMA) and immunohistochemistry (IHC)

The tissue microarrays (TMA) were made using buffered formalin-fixed and paraffin-embedded tissue sections from all the 106 pCCA patients according to our previous report [26]. Histological features of all samples were confirmed by hematoxylin and eosin (HE) staining before IHC. Core tissues with a 1.5-mm diameter were used for TMA construction.

For IHC, the slides were submerged in EDTA (pH = 9) buffer for optimal antigen retrieval. Primary HMGA1 antibody (1:100) or TRIP13 antibody (1:50) was applied and incubated with the specimens at 4 °C overnight. A biotin-labeled goat anti-rabbit antibody (Zsbio, Beijing, China) was applied for 30 min at room temperature. Subsequently, the slides were incubated with conjugated horseradish peroxidase streptavidin. The peroxidase reaction was developed using a 3,3-diaminobenzidine (DAB) solution (Zsbio). The IHC results screened using a TMA scanner (Pannoramic MIDI; 3DHISTECH, Budapest, Hungary) were evaluated independently by two senior pathologists and quantified using Quant Center Software. Quantitative IHC results were comprised of the score for staining intensity and the percentage of stained cells. The staining intensity score was defined as negative (0), weak (1), medium (2), or strong (3). The IHC score generated by Quant Center software was the sum of the product of the score for staining intensity multiplied by the percentage of stained cells respectively, according to previous studies [27]. The cohort was divided into low and high expression levels according to the cut-off values, which were identified as the point with the highest sum of the specificity and sensitivity in the receiver operating characteristic curve [9, 28].

### Quantitative real-time reverse transcription polymerase chain reaction (qRT-PCR)

Total RNA from fresh tissues/cells was extracted using TRIzol reagent (Invitrogen, Waltham, MA, USA), and cDNA was synthesized by reverse transcription and subjected to qRT-PCR using a kit from Toyobo (Osaka, Japan). SYBR Green Master Mix (Toyobo) and a StepOnePlus system (Applied Biosystems, Waltham, MA, USA) were used in this experiment. Glyceraldehyde 3-phosphate dehydrogenase (*GAPDH*) was used as an internal control. The primers used in this study are listed in Supplementary Table 2.

### Transfection and stable cell lines

HMGA1 and TRIP13 short-hairpin RNAs (shRNAs) were constructed using the lentivirus vector LV-5 (GenePharma). The virus particles were harvested 72 h after co-transfection with the packaging plasmids pGag/pol, pRev, and pVSV-G into HEK-293 T cells using RNAi-Mate and used to infect pCCA cells with polybrene (multiplicity of infection: 40) to generate corresponding stable cell lines (GenePharma). Full-length HMGA1 and TRIP13 sequences were ligated into the lentivirus vectors LV-GV492 and LV-GV365, respectively (Genechem). Transient transfection of small interfering RNA (siRNA) was realized with Lipofectamine 2000 (Thermo Fisher Scientific, Waltham, MA, USA). FBXW7 siRNAs were purchased from Biosune (Shanghai, China), and scrambled sequence was used as a control. Stable cell lines with HMGA1 overexpression or knockdown and with TRIP13 knockdown were selected with 4 μg/mL puromycin incubation for 7 days. The related sequences are listed in Supplementary Table 3. The efficiency of HMGA1/TRIP13/FBXW7 overexpression or knockdown was assessed using Western blot.

### Western blot and analysis

Total protein lysates were extracted from tissues or cultured cells using RIPA buffer (phenylmethylsulfonyl fluoride/RIPA [1:100]) and used for Western blot. The protein concentrations were determined using a BCA Protein Assay Kit (Tiangen Biotech, Beijing, China). Briefly, equal amounts of proteins (40 μg) were loaded onto gels, separated by sodium dodecyl sulfate polyacrylamide gel electrophoresis on 10% gels, and transferred to polyvinylidene difluoride membranes. Membranes were blocked with 5% nonfat milk and then incubated with the anti-HMGA1 (1:10000), anti-TRIP13 (1:200), anti-FBXW7(1:1000), or anti-epithelial-mesenchymal transition (EMT) antibodies (1:1000) overnight at 4 °C. Membranes were washed with TBST and incubated with secondary antibodies at 37 °C for 1 h. Finally, protein levels were confirmed and normalized using anti-GAPDH/laminB antibodies.

### Cell proliferation and colony formation assays

Cell proliferation assays were performed using a Cell Counting Kit 8 (CCK-8; Dojindo, Japan) according to the manufacturer’s instructions. For colony formation assays, 1000–1500 pCCA cells were seeded into each well of a 6-well plate and cultured in Dulbecco’s modified Eagle’s medium (DMEM) containing 10% fetal bovine serum (FBS) for 14 days. After fixation with methanol and staining with 0.1% crystal violet, the number of clones was counted under an inverted microscope.

### Wound healing assay

For wound healing assays, treated pCCA cells were cultured in 6-well plates at a density of 3 × 10^5^ cells/well until reaching confluence. A wound was then created in the center of the cell monolayers using a sterile pipette tip. Phase contrast images were captured at different times, and the percent wound healing was calculated as follows: healed migrated cell surface area/wound total surface area × 100%. Image J software was used.

### Cell migration and invasion assays

For migration assays, 5–10 × 10^4^ pCCA cells were seeded in 200 μL of 3% serum medium into the top chambers of inserts (BD Biosciences), and 600 μL medium with 20% FBS was added to the lower chamber. After incubating for 24–36 h, cells were fixed with 20% methanol for 30 min and stained with 0.1% crystal violet for 1 h. The cells on the upper surface were removed with a cotton swab, and the cell number in 10 random fields was counted from the lower surface. For invasion assays, the upper compartment of the chambers was precoated with 50 μL Matrigel [29].

### Soft agar colony forming assay

For three-dimensional (3D) sphere culture, soft agar colony formation assays were used. Briefly, low-melting-temperature agarose containing 10% FBS and DMEM was diluted to a final concentration of 0.6% and solidified at 4 °C in wells of a 6-well plate [30]. Next, 10,000 cells/well were immediately plated in the top layer of low-melting-temperature agarose containing 10% FBS at a final concentration of 0.3%. Cells were covered with 1 mL DMEM per well and incubated at 37 °C with 5% CO_2_ for 1 week. Medium was changed every other day, and cells were imaged. Colonies were counted using an Olympus IX81 inverted microscope. At least three independent experiments were performed in triplicate.

### Xenograft models

Female BALB/c nude mice (5 weeks of age) were purchased from GemPharmatech Company (Nanjing, China). Stable clones of pCCA (QBC939) cells, transfected with shHMGA1, HMGA1, HMGA1/shTRIP13, or the control vector, were subcutaneously injected into the right flanks of nude mice (*n* = 6/group). Tumor diameters were measured with an external caliper every 3 days as previously described (Z et al., 2019).

For in vivo hepatic metastasis assays, 5 × 10^5^ treated QBC939 cells were injected into the caudal vein of nude mice (n = 6/group). Mice were sacrificed after 5 weeks and examined for hepatic metastases. Optical and pathological images were collected to visualize primary tumor growth and metastatic lesion formation. Tumor metastasis was finally confirmed with HE staining by the criteria including tissue atypia (abnormal tissue arrangement, cell morphology), and nuclear atypia (large nuclear, increased mitosis,etc.).

All nude mice were maintained under specific pathogen-free conditions in the Experimental Animal Department of Shandong University. All animal experiments were approved by the Clinical Research Ethics Committees of Shandong University.

### RNA-seq and computational analyses

RNA-seq was performed to detect the mRNA expression profiles of TRIP13-silenced pCCA cells at GenePharma (China) using the Illumina HiSeq 2500 platform (LC Sciences, Hangzhou, China). HISAT package was used to align the reads to the genome and generate raw counts corresponding to each known gene (32,331 genes), and String Tie was used to evaluate the expression levels of mRNAs by calculating fragments per kilobase million (FPKM). Differentially expressed genes were selected with fold change>4 and Padj< 0.10, and gene ontology (GO) analysis was used for pathway enrichment with Cytoscape (ClueGo) for data.

### Luciferase reporter assay

QBC939 cells (5 × 10^4^ cells/well) were seeded in 24-well plates in triplicate and allowed to attach for 24 h. Cells were then transiently transfected with the indicated plasmids and the pRL-TK Renilla luciferase plasmid using Lipofectamine 2000 (Invitrogen). Forty-eight hours after transfection, the cells were harvested and processed using a Dual Luciferase Reporter Assay Kit (Promega, Madison, WI, USA) according to the manufacturer’s instructions. Luciferase activity was evaluated using a Dual-Luciferase Reporter Assay System (Beyotime) with Renilla luciferase as internal control to eliminate the chaos of transfection efficiency. The target DNA fragment genes (HMGA1/TRIP13/c-Myc) were cloned into the pcDNA3.1 or pGPU6 vector. The empty pcDNA3.1 basic vector was used as the negative control, and the pcDNA3.1 promoter vector, containing the gene of *TRIP13,FBXW7 or HMGA1* promoter upstream of the luciferase gene respectively, was used as the positive control. The human promoter regions generated by PCR-amplification, were cloned into the *Kpn*I/*Hind*III sites of the PGL3-basic dual luciferase reporter plasmid to generate *TRIP13,FBXW7 or HMGA1* luciferase reporters. The reporter gene activity was determined by normalization of the firefly luciferase activity to Renilla luciferase activity. The promoter region sequences of HMGA1/TRIP13/c-Myc were provided in Supplementary Table 4.

### Statistical analysis

SPSS 17.0 and GraphPad Prism 5 software package version 5.03 (GraphPad Prism Software, San Diego, CA, USA) were used to perform statistical analyses. The χ^2^ test was used to assess the correlations between HMGA1/TRIP13 and clinicopathological factors. The Kaplan-Meier method and log-rank test were used to determine cumulative overall survival rates and survival curves, respectively. The independent prognostic factors were analyzed by multivariate analysis with the Cox-regression model. T-test, one- or two-way analysis of variance (ANOVA) were used for statistical comparisons between groups. Results with *P* values of less than 0.05 were considered significant.

## Results

### Expression and clinical significance of HMGA1 in pCCA

**Fig. 1:**
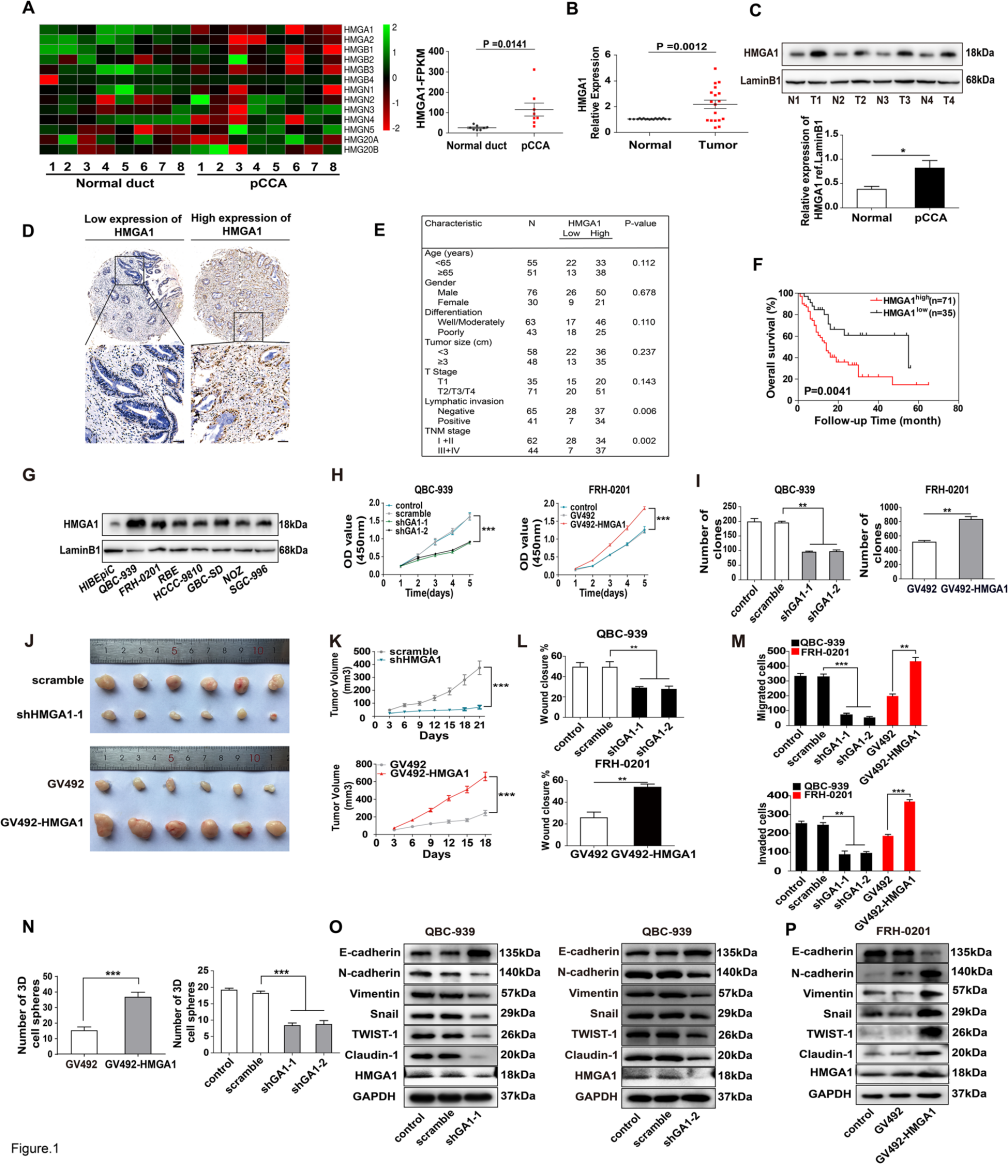
HMGA1 enhanced oncogenic progression of pCCA cells. **a** Left: Heatmap of HMG gene expression profiles in eight pairs of pCCA tissues and adjacent normal tissues. Right: FPKM of HMGA1 in pCCA and adjacent normal tissues were shown. Data were were analyzed by paired t tests (**b**) *HMGA1* mRNA expression in 18 paired pCCA and adjacent normal duct tissues, detected by qRT-PCR. *P* value was calculated with paired t tests. **c** Up: HMGA1 expression in four randomly-selected pairs of pCCA tissues (T) and adjacent normal tissues (N), detected by Western blot. Bottom: quantification of bands in the left panel. **d** Representative images of immunohistochemical staining for low/high expression of HMGA1 in the tissue microarray (top: 100× magnification, scale bar, 200 μm; bottom: 400× magnification, scale bar, 50 μm). **e** Correlation between HMGA1 expression and clinicopathological features (Chi-square tests). **f** Kaplan-Meier survival analysis in patients with pCCA with low (*n* = 35) and high (*n* = 71) HMGA1 expression (cut-off: 77.4). **g** HMGA1 expression in human cholangiocarcinoma cell lines, gallbladder carcinoma cell lines, and intrahepatic bile duct cell line HiBEpiC. (H) *HMGA1* was silenced in QBC939 and overexpressed in FRH0201, and the effects of HMGA1 on pCCA cell proliferation were measured by CCK-8 assays. **i** effects of HMGA1 on colony formation in QBC939 and FRH0201 cells after knockdown in QBC939 or overexpression in FRH0201. **j** QBC939 cells with stable *HMGA1* knockdown (up) or overexpression (bottom) were subcutaneously injected to nude mice for xenograft model (*n* = 6/group). **k** Tumor volumes in xenografts established using cells infected with lentivirus carrying scrambled shRNA, shHMGA1, empty vector, or GV492-HMGA1. **l**,**m** The effects of HMGA1 on migration and invasion were detected with wound-healing assay(**l**) and transwell assay(**m**) after silencing *HMGA1* in QBC939 or overexpressing *HMGA1* in FRH0201 cells. **n** Effects of HMGA1 on stemness was detected by 7-day-sphere formation activity with stable *HMGA1*-silencing or *HMGA1*-overexpressing QBC939 cells. **o**,**p** Effects of HMGA1 on EMT were detected by Western blot after silencing *HMGA1* in QBC939(**o**) and overexpressing *HMGA1* in FRH0201 cells(**p**). *P < 0.05, **P < 0.01, and ***P < 0.001 compared with the corresponding control group. Data are shown as means ±S.D., and statistical significance was analyzed with one-way ANOVA(**h** and **k**) or T-test. Analyzed data were from three independent experiments, and each subgroup was performed at least in triplicate (**c**,**g**, **h**,**i**, **l**-**p**)

Transcriptome sequencing profiles in eight pCCA tissues and paired tumor-adjacent bile duct tissues were used to identify differentially expressed genes in pCCA (BioProject accession PRJNA517030 and PRJNA547373). According to the criteria set as fold change> 4 and Padj<0.10, 180 genes were selected (Supplemental Table 5). Among the HMG family, only *HMGA1* was significantly upregulated in pCCAs compared with adjacent bile duct tissues (Fig. 1a). Moreover, qRT-PCR with 18 pCCA pairs (Fig. 1b) and WB with four pCCA pairs further confirmed HMGA1 upregulation in pCCA (Fig. 1c). In a retrospective cohort of patients with pCCA radical resection (*n* = 106), the expression and localization of HMGA1 were detected with IHC. HMGA1 was mainly expressed in the nucleus (Fig. 1d), consistent with its function as a transcription factor.

Moreover, high expression of HMGA1 was significantly associated with positive lymphatic invasion and advanced TNM stage (Fig. 1e), indicating that HMGA1 may promote the invasion of pCCA. In overall survival curves, high HMGA1 expression was correlated with unfavorable prognosis (Fig. 1f; Supplementary Table 6), suggesting that HMGA1 may be a prognostic biomarker of pCCA. Cox-regression hazard models confirmed that HMGA1 tended to be an independent prognostic biomarker of pCCA, but the statistical significance is not that notable (*P* = 0.072) (Supplementary Table 6).

### HMGA1 promoted the proliferation, invasion, stemness, and EMT in pCCA cells

Next, we measured HMGA1 expression in various biliary cell lines including normal biliary epithelium cell line HIBEpiC, the pCCA cell lines QBC-939 and FRH-0201, iCCA cell lines RBE and HCCC-9810, gallbladder carcinoma cell lines GBC-SD, NOZ and SGC-996. HIBEpiC cells showed significantly lower HMGA1 expression, whereas the expression of HMGA1 was increased in all these cell lines (Fig. 1g, Supplementary Figure 1A). In both QBC-939 and FRH-0201 cells, *HMGA1* was silenced with two independent shRNAs or overexpressed with a lentivirus carrying the *HMGA1* cDNA (Supplementary Figure 1B and 1C). CCK8 and colony formation assays demonstrated that *HMGA1* knockdown significantly impaired proliferation, whereas *HMGA1* overexpression promoted pCCA proliferation (Fig. 1h and i, Supplementary Figure 2A). Stable QBC-939 cells with *HMGA1* knockdown or overexpression were injected subcutaneously to establish xenografts in mice. The tumor volume and weight of xenografts were extensively decreased by HMGA1 knockdown and increased by HMGA1 overexpression (Fig. 1j and k, Supplementary Figure 2B). In addition, wound healing and transwell assays demonstrated that HMGA1 promoted the migration and invasion of QBC-939 and FRH-0201 cells (Fig. 1l and m, Supplementary Figure 2C and D). All above results indicated that HMGA1 had extensive influences on pCCA progression including proliferation, migration and invasion.

To explain this multiple functions of HMGA1 on pCCA progression, the effects of HMGA1 on cell stemness and EMT were investigated because previous study suggested HMGA1 is an important factor involved in cell stemness and EMT [13]. Sphere-formation assays showed that *HMGA1* upregulation increased pCCA stemness, whereas HMGA1 downregulation suppressed stemness (Fig. 1n, Supplementary Figure 2E). Moreover, E-cadherin expression was decreased, and other EMT biomarkers including N-cadherin, Vimentin, Snail, Twist-1, and Claudin-1, were upregulated following *HMGA1* overexpression, and downregulated following *HMGA1* knockdown (Fig. 1o and p). These results indicated that HMGA1 played important roles in stemness and EMT of pCCA, which thus influenced proliferation, migration and invasion.

### HMGA1 promoted the transcription and expression of TRIP13

**Fig. 2:**
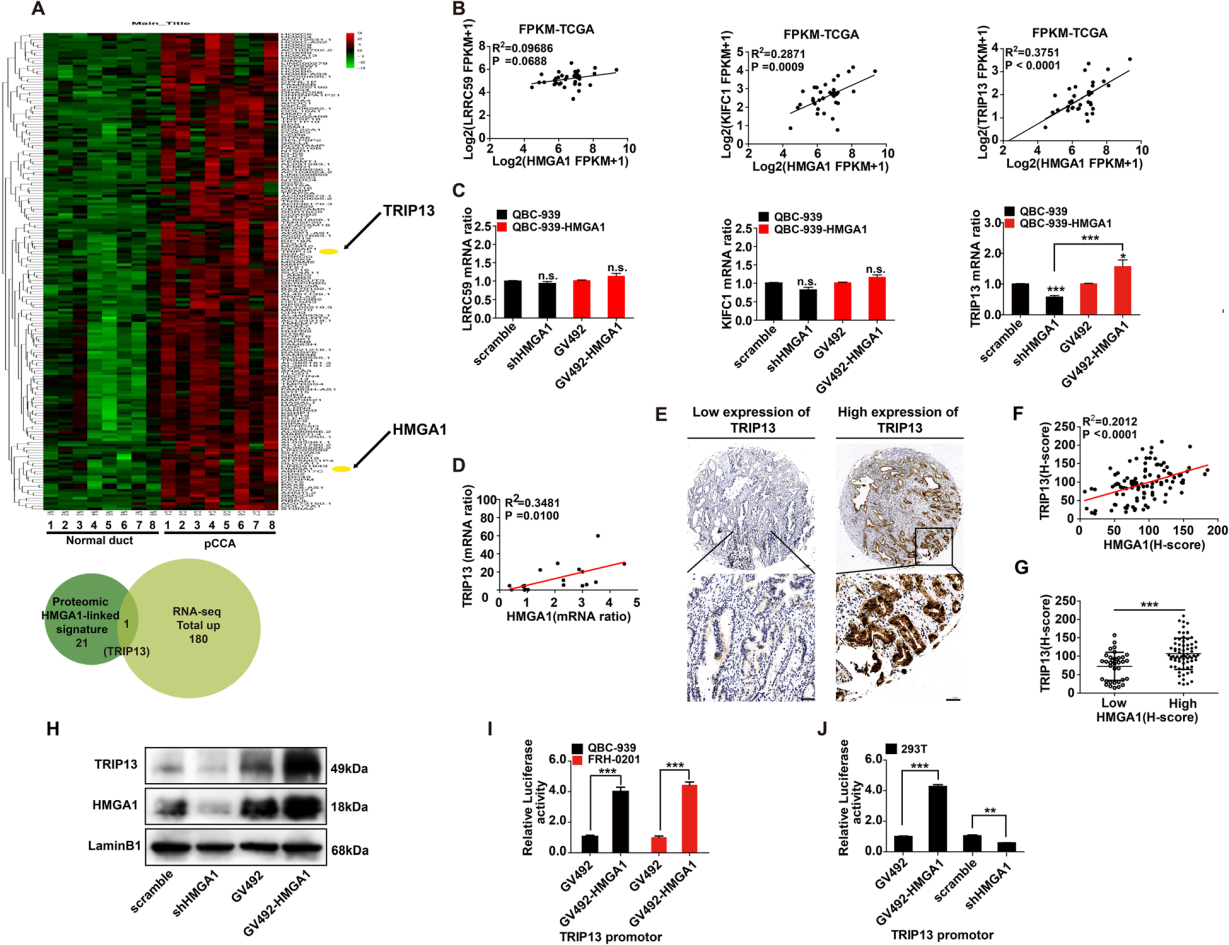
HMGA1 promoted the transcription and expression of *TRIP13*. **a** Heatmap and hierarchical clustering analysis revealed 180 up-regulated gene expression profiles in eight pairs of pCCA tissues and adjacent normal tissues. TRIP13 was filtered out as the only overlapping gene between the 21 Proteomic HMGA1-linked signature and 180 total upregulated genes (**a**). **c** Correlations between *HMGA1* and *LRRC59/KIFC1/TRIP13* mRNA in 36 cases of CCA were evaluated. The linear correlations were analyzed using the Spearman method. **d** Changes in *LRRC59*/*KIFC1*/*TRIP13* mRNA after knocking down or overexpressing HMGB1 in QBC939 cells. **e** Correlation between *HMGA1* and *TRIP13* mRNA in 12 fresh pCCA tissues. mRNA ratio was calculated by mRNA (tumor)/mRNA (adjacent tissue). **f** Representative IHC images of low and high TRIP13 expression (top: 100× magnification, scale bar, 200 μm; bottom: 400× magnification, scale bar, 50 μm). **g** IHC scores of HMGA1 were significantly associated with TRIP13 scores in pCCA tissues. **h** Patients with high HMGA1 expression had higher TRIP13 expression. **i** TRIP13 expression was detected in stable QBC939 cells with *HMGA1* knockdown or overexpression. **j** and **k** HMGA1 promoted the transcription of *TRIP13* in both QBC939 and FRH0201 cells (**j**), and 293 T cells(**k**). The transcriptional activity of *TRIP13* was detected with luciferase assays. * represented *P* < 0.05, and *** represented *P* < 0.001 compared with control or indicated groups, calculated by T-test in (**j**, **h** and **k**). Spearman correlation analysis was performed in (**c**, **e** and **g**). Analyzed data were from three independent experiments, and shown as means± SEM

In mRNA sequencing of eight pairs of pCCA and normal bile duct tissues, 180 genes were up-regulated (Fig. 2a, Supplementary Table 5). In previous study, a total of 21 proteomic signatures regulated by HMGA1 in breast cancer were reported, and three of them (*KIFC1*, *LRRC59*, and *TRIP13*) were verified to promote breast cancer progression [31]. Interestingly, *TRIP13*, was identified by both our mRNA sequencing and previous proteomic HMGA1-linked signatures (Fig. 2a, Supplementary Table 5). The mRNA levels of *KIFC1*, *LRRC59*, and *TRIP13* were evaluated using 36 cases of CCA from The Cancer Genome Atlas (TCGA) database (https://tcga-data.nci.nih.gov/tcga/), and their correlations with HMGA1 were analyzed (Fig. 2b). *KIFC1* and *TRIP13* showed positive correlation with HMGA1 of the 36 CCAs, but qRT-PCR showed that only *TRIP13* expression was regulated by HMGA1 in QBC-939 (Fig. 2c). Our qRT-PCR results with 18 pCCA tissues also supported the strong positive correlation between *HMGA1* and *TRIP13* (Fig. 2d). TRIP13 expression was detected by IHC in 106 cases in pCCA TMA (Fig. 2e). The IHC score of TRIP13 was significantly associated with the IHC score of HMGA1(Fig. 2f), and patients with high HMGA1 expression had high TRIP13 expression (Fig. 2g). In QBC-939 cells, regulation of HMGA1 expression led to corresponding changes of TRIP13 (Fig. 2h). Finally, luciferase assays demonstrated that HMGA1 promoted the transcription of *TRIP13* in pCCA cells (Fig. 2i) and 293 T cells (Fig. 2j). All above results suggested that HMGA1 induced *TIRP13* expression via promoting its transcription.

### TRIP13 promoted cancer progression and was correlated with poor prognosis in pCCA

**Fig. 3:**
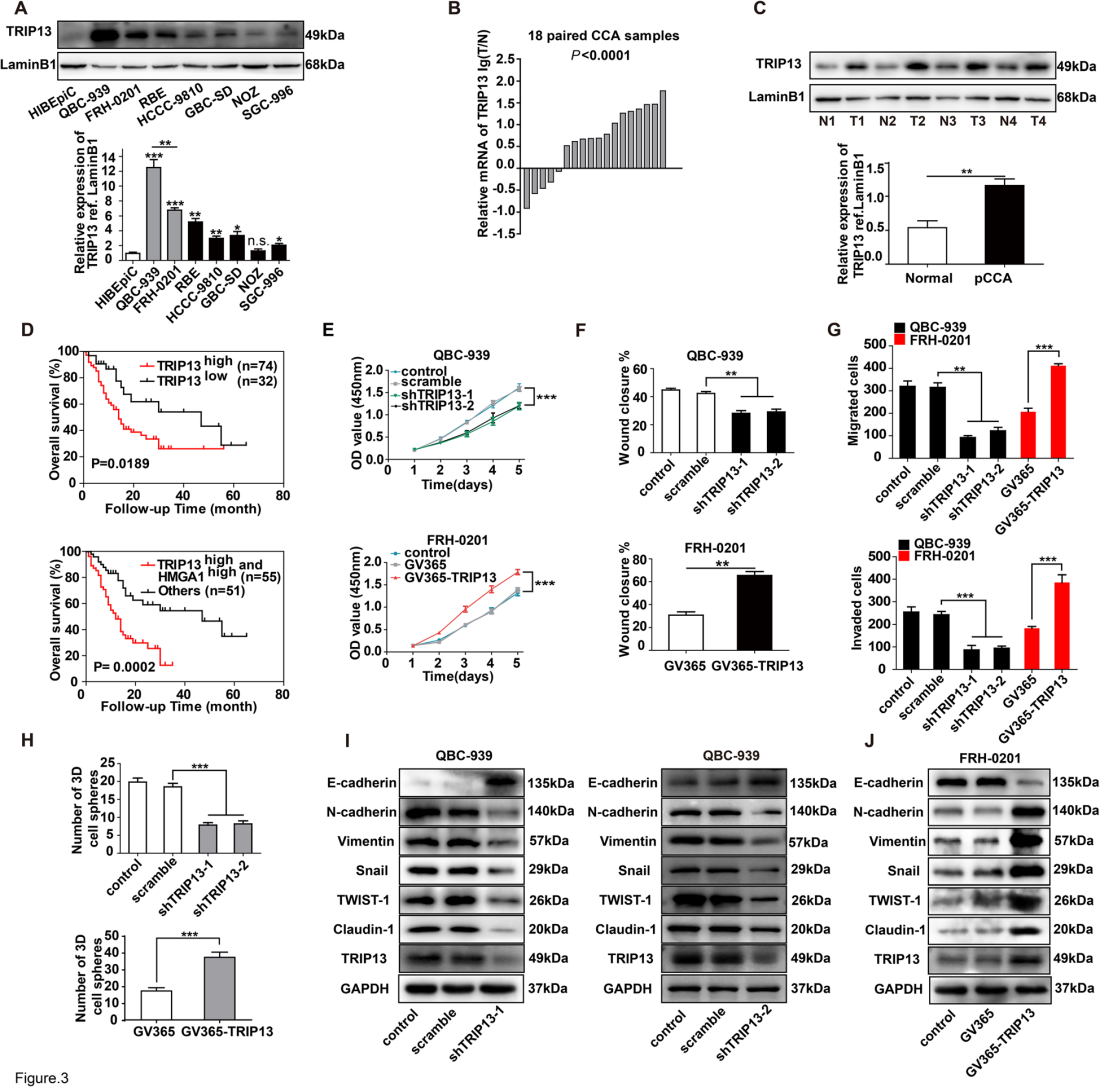
TRIP13 was correlated with poor prognosis and progression of pCCA. **a** TRIP13 expression in biliary cell lines. Up: Expression of TRIP13 in HiBEpiC, iCCA cell lines, pCCA cell lines and gallbladder cancer cells. Bottom: the quantification of the TRIP13 bands in WB. **b** *TRIP13* mRNA levels in 18 pairs of pCCAs and adjacent normal bile duct tissues were detected with qRT-PCR, and showed as log (*TRIP13*^Tumor^/*TRIP13*^Non-tumor^). **c** TRIP13 expression was detected in four pairs of pCCA tissues and adjacent normal duct tissues with WB (up), and the bands were quantified (bottom). **d** The prognostic significance of TRIP13 expression (up), and co-expression of TRIP13 and HMGA1(bottom) were analyzed with survival analysis by log-rank test. **e** *HMGA1* was knocked down in QBC-939 cells and overexpressed in FRH-0201 cells; cell proliferation was detected with CCK8 assays within 4 days of culture. **f**,**g** Migration and invasion of QBC-939 and FRH-0201 cells, as detected by wound healing assay (**f**) or transwell assay(**g**) after silencing or overexpressing *HMGA1*. **h** 7-day sphere formation assays were performed to determine the effects of TRIP13 on stem cell characteristics of pCCA cells. **i**, **j** Effect of TRIP13 on the EMT in pCCA cells was evaluated by WB after silencing (**i**) or overexpressing (**j**) TRIP13 expression. *, ** and *** represents *P* < 0.05, *P* < 0.01 and *P* < 0.001, compared with the corresponding control group, analyzed with the T-test (**c**,**f**-**h**) or one-way ANOVA (**e**). Data were from three independent experiments and shown as means ± S.E.M.

TRIP13 expression was highest in QBC-939 cells among the detected biliary cell lines including HIBEpiC, QBC-939, FRH-0201, RBE, HCCC-9810, GBC-SD, NOZ and SGC-996 (Fig. 3a). Evaluation of TRIP13 expression by qRT-PCR in 18 pairs of pCCA tissues and WB in four pairs of pCCA tissues demonstrated that TRIP13 was upregulated (Fig. 3b and c). In the validation cohort, patients with high expression of TRIP13 had poorer prognoses than those with low expression(*P* = 0.019) (Fig. 3d). Intriguingly, high expression of both TRIP13 and HMGA1 was a more sensitive prognostic factor than TRIP13 or HMGA1 alone (*P* = 0.0002) (Fig. 3d). Multivariate analysis also identified TRIP13 as an independent prognostic biomarker of pCCA (hazard ratio = 1.95, *P* = 0.046; Supplementary Table 6). Importantly, TRIP13 was significantly associated with TNM stage and tended to be associated with lymphatic invasion, similar to HMGA1 (Supplementary Table 7). CCK8 assays demonstrated that TRIP13 promoted the proliferation of pCCA cells (Fig. 3e). Wound healing and transwell assays suggested that TRIP13 was required in pCCA cell migration and invasion (Fig. 3f and g, Supplementary 3A and B). Similar to HMGA1, TRIP13 was essential for stemness and the EMT of pCCA cells (Fig. 3h-j, Supplementary 3C). Taken together, these results suggested that TRIP13 promoted the progression of pCCA.

### TRIP13 was required in HMGA1-induced pCCA progression

**Fig. 4:**
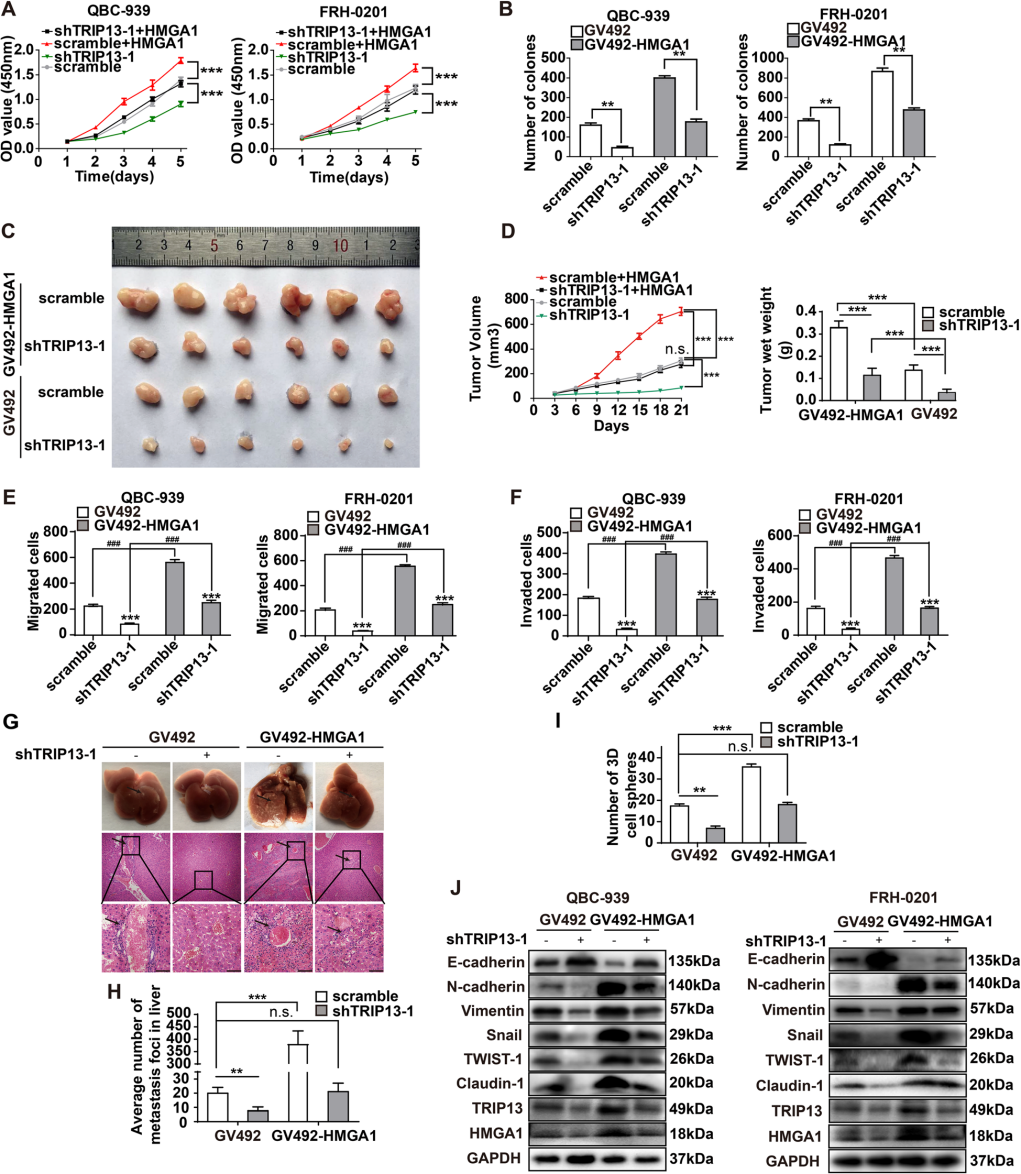
TRIP13 was required for HMGA1-induced pCCA progression. **a**,**b** CCK8 assay (**a**) and colony formation assay (**b**) showed that *TRIP13* knockdown attenuated the proliferation induced by *HMGA1* overexpression. **c** Stable QBC-939 cells with/without *HMGA1* overexpression and *TIRP13* knockdown were injected subcutaneously for xenografts. **d** *TIRP13* knockdown significantly decreased the volume and weight of xenografts. **e**,**f** *TIRP13* knockdown attenuated cell ability of migration(**e**) and invasion(**f**) of pCCA cells. **g** TRIP13 is required in HMGA1-induced metastasis. Up: metastasis of stable QBC-939 cells with *TIRP13* knockdown and/or *HMGA1* overexpression to the liver, as verified by HE staining. The mouse metastasis model was established by tail vein injection of stable cells. Scale bar: 50 μm. **h** numbers of metastatic lesions on the surface of the liver. **i** 7-day sphere formation assays were performed to evaluated the effects of TRIP13 on HMGA1-induced pCCA stemness. **j** EMT biomarkers were detected to evaluate the effects of TRIP13 on HMGA1-induced pCCA EMT. n.s. represents not significant. *, ** and *** represents *P* < 0.05, *P* < 0.01 and *P* < 0.001, analyzed with one-way ANOVA(**a**,**d**) or T-test (**b**,**e**,**f**,**i**,**h**,**j**). Analyzed data were from three independent experiments, and each subgroup was performed at least in triplicate (**a**,**b**, **e**,**f**,**i**,**j**)

To detect the role of TRIP13 in HMGA1-induced pCCA progression, *TRIP13* was silenced in HMGA1-overexpressing cells. CCK8 and colony formation assays demonstrated that TRIP13 knockdown attenuated HMGA1-induced proliferation in QBC-939 and FRH-0201 cells (Fig. 4a and b, Supplementary Figure 4A). Xenografts were established with HMGA1-overexpressing stable cells with or without *TRIP13* knockdown, showing that *TRIP13* knockdown significantly reduced tumor volume and weight which were increased by *HMGA1* overexpression (Fig. 4c and d). The migration and invasion of HMGA1-overexpressing QBC-939 and FRH-0201 cells were impaired after *TRIP13* knockdown (Fig. 4e and f, Supplementary Figure 4B). Stable QBC-939 cells with *HMGA1* overexpression and/or *TRIP13* knockdown were injected into the tail vein, and metastases to the liver was detected with HE staining (Fig. 4g). *HMGA1* overexpression increased the number of metastatic lesions, whereas silencing of *TRIP13* neutralized this effect (Fig. 4h). 3D sphere formation and EMT biomarker expression showed that *TRIP13* knockdown significantly impaired HMGA1-induced cell stemness and the EMT (Fig. 4i and j, Supplementary Figure 4C). All these results indicated that HMGA1 promoted the stemness and EMT by elevating TRIP13 expression in pCCA cells.

### FBXW7 suppressed TRIP13-induced progression by degrading c-Myc

**Fig. 5:**
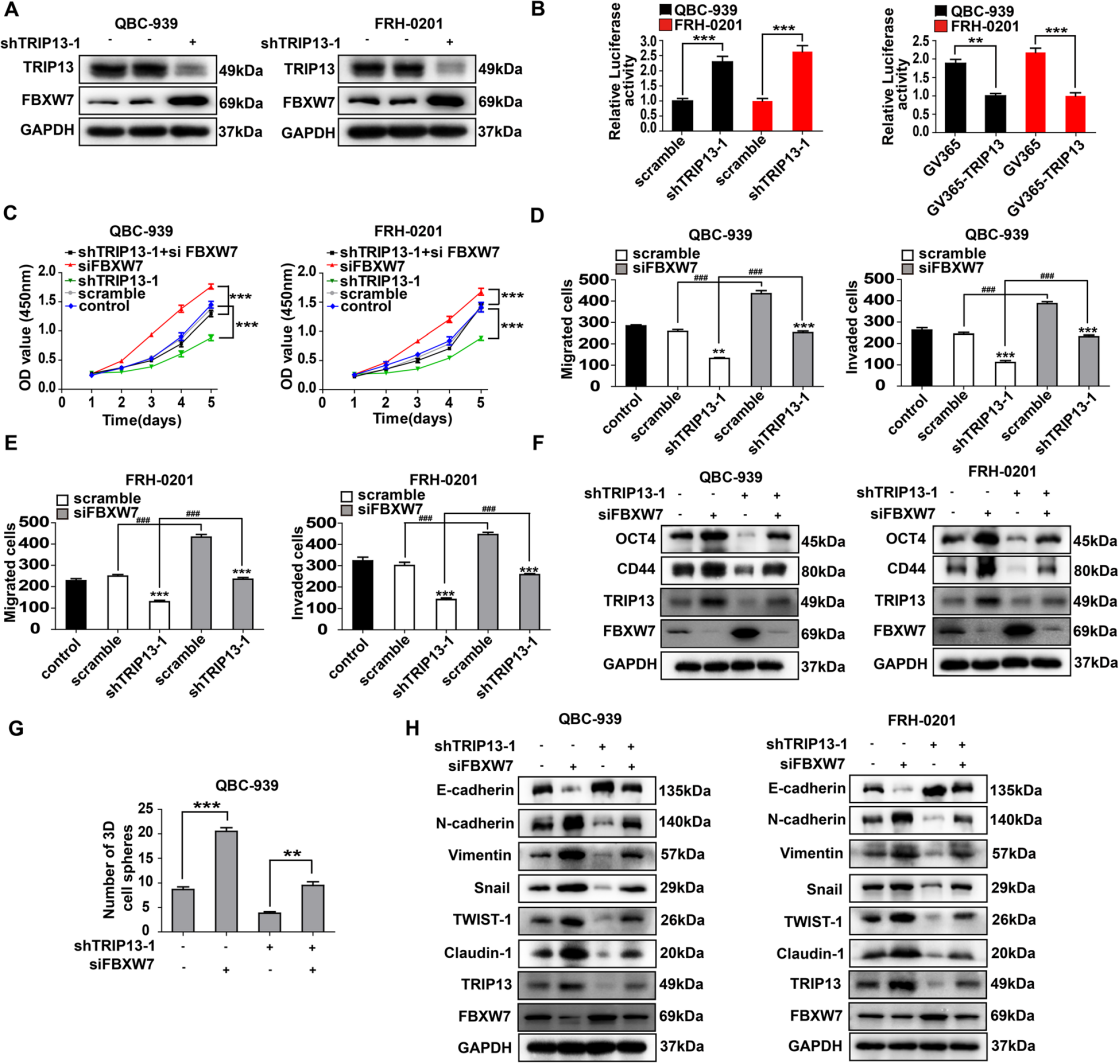
FBXW7 suppressed TRIP13-induced stemness and EMT. **a** *TRIP13* knockdown decreased FBXW7 expression in QBC-939 and FRH-0201 cells. **b** Luciferase assays showed that TRIP3 inhibited *FBXW7* transcription in QBC-939 and FRH-0201. **c** Silencing *TRIP13* suppressed pCCA proliferation and knocking down *FBXW7* had contrary effects. **d**,**e** Transwell assay showed that silencing *FBXW7* promoted the migration(**d**) and invasion(**e**) which was decreased by *TRIP13* knockdown. **f**,**g** QBC-939 and FRH-0201 were transfected with shTRIP13 and/or siFBXW7. The biomarkers of stemness (CD44 and OCT4)(**f**) and 7-day sphere formation assay(**g**) were performed. (H) EMT biomarkers were detected after regulating TRIP13 and/or FBXW7 expression in QBC939(left) or FRH0201(right). In (**b**,**d**,**e**,**g**), ** and *** represents *P* < 0.01 and < 0.001, compared with corresponding control group or indicated groups. In (**b**,**d**), the *P* value was calculated with T-test(**b**,**d**,**g**) or one-way ANOVA(**c**). Analyzed data were from three independent experiments, and each subgroup was performed at least in triplicate

We previously reported that F-box/WD repeat-containing protein 7(FBXW7) suppressed the stemness and EMT of CCA [32], and a recent study proposed that FBXW7 expression was inhibited by TRIP13 in glioblastoma [33], so we further investigated the correlation between FBXW7 and TRIP13 in pCCA progression. WB, qPCR and luciferase assay showed that *TRIP13* knockdown significantly increased the transcription and expression of *FBXW7* in both QBC-939 and FRH-0201(Fig. 5a and b, Supplementary Figure 5A and B). *TRIP13* and *FBXW7* mRNA levels in the HMGA1-silenced and HMGA1-overexpressed xenografts (Fig. 1j and Fig. 4c) were detected with qRT-PCR, reflecting that TRIP13 and FBXW7 are downstream effectors of HMGA1 (Supplementary Figure 6). Moreover, the *FBXW7* knockdown facilitated the proliferation, migration and invasion of pCCA cells, which was attenuated by *TRIP13* knockdown (Fig. 5c-e, Supplementary Figure 5C). Biomarkers of stemness or EMT, and sphere formation assay were performed after silencing *TRIP13* or *FBXW7* using WB and qRT-PCR (Fig. 5f-h Supplementary Figure 5D and E). Consequently, *TRIP13* knockdown attenuated stemness and EMT of pCCA cells, while *FBXW7* knockdown reversed this tendency, indicating that FBXW7 was involved in the TRIP13-induced stemness and EMT.

**Fig. 6:**
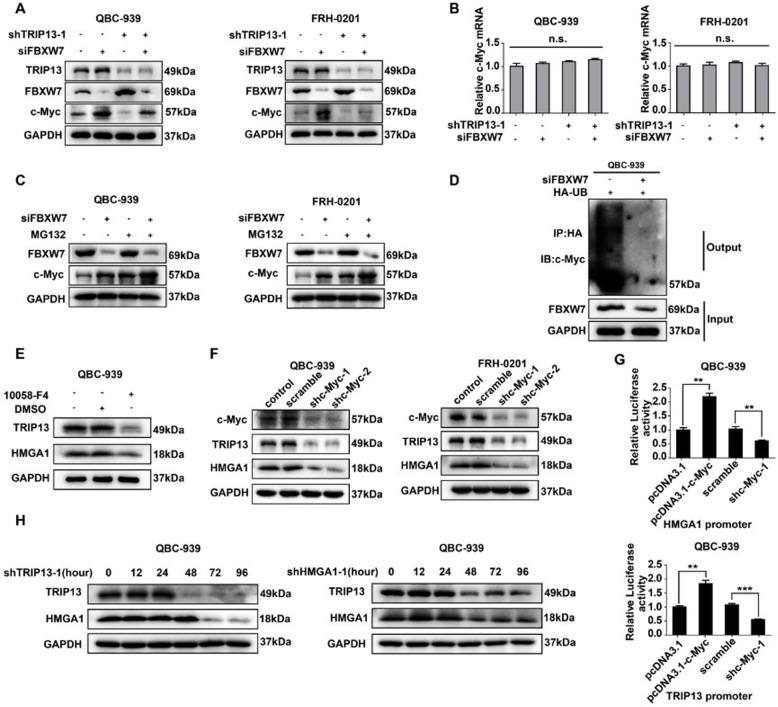
HMGA1-TRIP13 axis promotes invasion, stemness and EMT in a positive feedback pathway dependent on c-Myc. **a**,**b** WB(**a**) and qRT-PCR(**b**) showed that *FBXW7* and *TRIP13* knockdown regulated c-Myc expression but had little effect on *c-Myc* mRNA. **c** MG132 inhibited the FBXW7-induced c-Myc degradation. QBC-939(left) and FRH-0201(right) were incubated in 10 μM MG132 for 12 h before lysis. **d** *FBXW7* knockdown decreased the ubiquitination of c-Myc. 24 h after transfection with HA-Ub and siFBXW7, QBC-939 cells were incubated in 10 μM MG132 for 12 h. HA beads were used to precipitate HA-interacting proteins and c-Myc antibody was used to detect the ubiquitinated c-Myc. **e**,**f** c-Myc inhibitor 10,058-F4(**e**) and *c-Myc* knockdown(**f**) decreased the expression of HMGA1 and TRIP13 in pCCA cells. 10,058-F4(10 μM) was used to pre-incubate QBC-939 cells for 12 h. **g** Luciferase assays revealed that c-Myc promoted the transcription of *TRIP13* and *HMGA1* of QBC-939 cells. The transcriptional activity of *HMGA1*(up) and *TRIP13*(bottom) were detected with luciferase assays. ** represents *P* < 0.01, calculated with T-test. **h** *HMGA1* knockdown rapidly decreased TRIP13 expression, while *TRIP13* knockdown attenuated HMGA1 expression 12–24 h later. QBC-939 were transfected with shTRIP13 or shHMGA1 and incubated for 0–96 h. Analyzed data were from three independent experiments, and each subgroup was performed at least in triplicate

*c-Myc* is a well-known onco-protein involved in stemness, and also a target of FBXW7 for ubiquitination. In QBC-939 and FRH-0201, c-Myc expression was elevated by *FBXW7* knockdown and reduced by *TRIP13* knockdown (Fig. 6a, Supplementary Figure 7A), but qRT-PCR implicated that *c-Myc* mRNA was not influenced (Fig. 6b), suggesting that c-Myc degradation, instead of transcription, was affected by TRIP13 and FBXW7. Incubation in the ubiquitination inhibitor MG132(10uM) extensively eliminated the FBXW7-induced degradation of c-Myc (Fig. 6c, Supplementary Figure 7B). Additionally, *FBXW7* knockdown substantially decreased ubiquitinated c-Myc in HA-Ubiquitin-overexpressing QBC-939(Fig. 6d). Collectively, TRIP13 promoted pCCA stemness and EMT by suppressing *FBXW7* transcription and thus stabilizing c-Myc.

### HMGA1-TRIP13 axis promotes stemness and EMT in a positive feedback pathway dependent on c-Myc

With the transcription-factor-predicting software (Jaspar software), we found that c-Myc was predicted to promote the transcription of *HMGA1* and *TRIP13* so we further investigated the role of c-Myc in HMGA1 and TRIP13 expression. WB (Fig. 6e and f) and qRT-PCR (Supplementary Figure 8A-C) showed that both 10,058-F4(a c-Myc inhibitor) and *c-Myc* knockdown significantly decreased expression of HMGA1 and TRIP13. Luciferase assay validated that *c-Myc* overexpression increased the transcription of *HMGA1* and *TRIP13*, and *c-Myc* knockdown had contrary effects (Fig. 6g). These results implicated that c-Myc was able to induce the transcription of *HMGA1* and *TRIP13*. Moreover, we knocked down *TRIP13* in QBC-939, and demonstrated that TRIP13 can also regulate HMGA1 expression (Fig. 6h). However, it was interesting to note that TRIP13 expression was decreased almost at the same time of *HMGA1* knockdown, while HMGA1 expression was attenuated about 24 h later than *TRIP13* knockdown (Fig. 6h). This may be explained by that TRIP13 regulated HMGA1 expression by stabilizing c-Myc, requiring more time than that HMGA1 directly regulated *TRIP13* transcription. Combined with previous results that HMGA1-TRIP13 axis stabilized c-Myc, we postulated that HMGA1 had a positive feedback loop to amplify its biological effect depending on c-Myc.

**Fig. 7:**
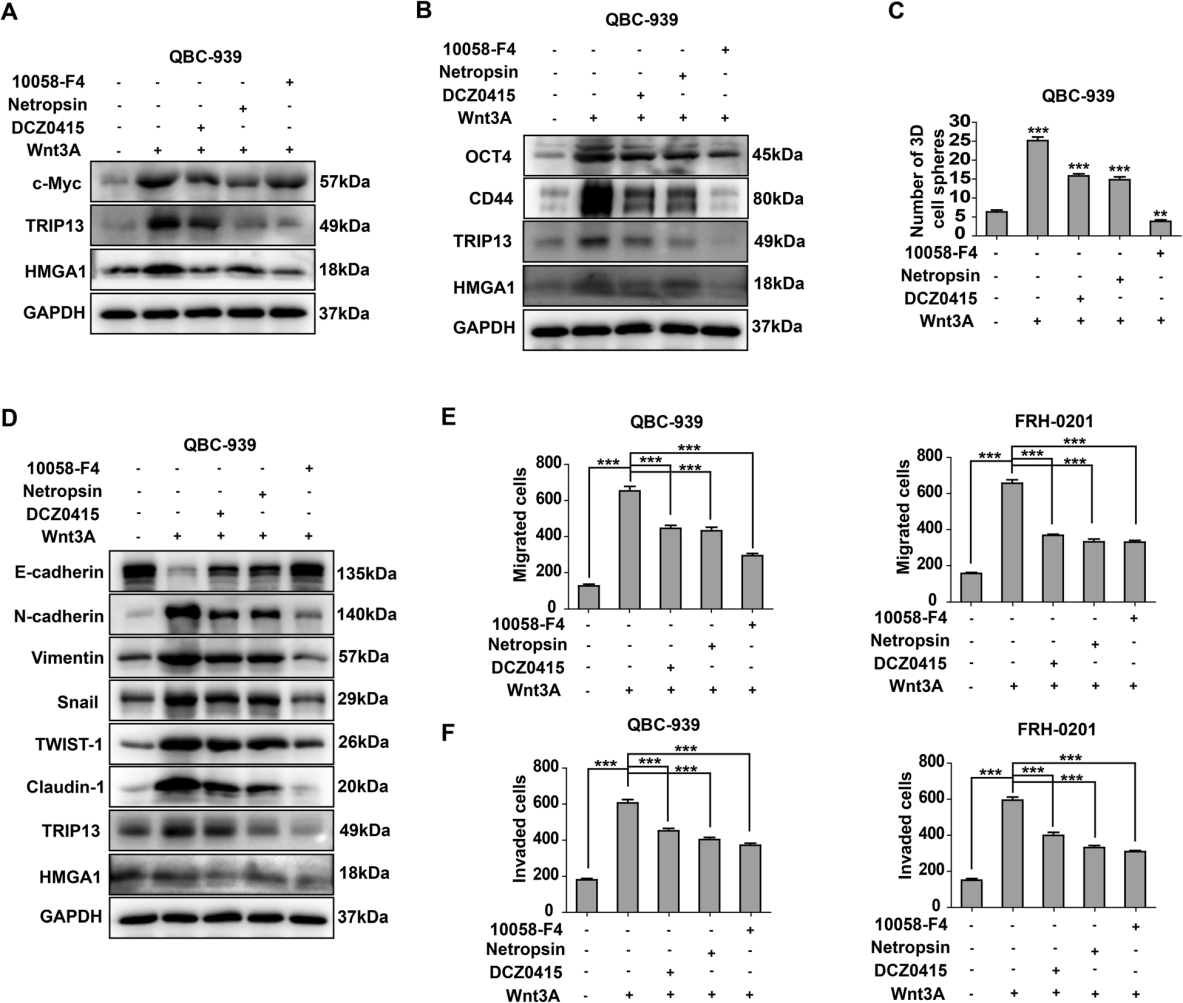
Inhibitors of HMGA1, TRIP13 or c-Myc can block their feedback and suppress pCCA progression. **a** In the presence of Wnt3A(100 ng/ml), 10 μM 10,058-F4, 10μg/ml HMGA1 inhibitor Netropsin and 10 μM TRIP13 inhibitor DCZ0415 were used to incubate QBC-939 for 12 h. All these inhibitors inhibited the expression of c-Myc, TRIP13 and HMGA1. **b**,**c** WB **b** and sphere formation(**c**) indicated the inhibitors of c-Myc, HMGA1 and TRIP13 decreased stemness of pCCA. **d** Inhibitors of c-Myc, HMGA1 and TRIP13 attenuated EMT of pCCA. **e**,**f** Inhibitors of c-Myc, HMGA1 and TRIP13 attenuated migration (**e**) and invasion (**f**) of pCCA in Wnt3a stimulation. ** and *** represents *P* < 0.01 and < 0.001, calculated with T-test. Analyzed data were from three independent experiments and displayed by the mean + S.E.M.

Wnt-β-catenin pathway is a well-accepted activator of c-Myc, so Wnt3a(100 ng/ml) was used to incubate QBC-939 for 12 h to stimulate Wnt signaling. The recently developed small-molecule inhibitor of TRIP13, DCZ0415(10uM) [34], HMGA1 inhibitor Netropsin (10μg/ml), and 10,058-F4(10uM) were used to incubate QBC-939 cells. Interestingly, any inhibitor can decrease the expression of c-Myc, HMGA1 and TRIP13(Fig. 7a, Supplementary Figure 7D), suggesting that these three factors were in the same positive feedback loop. Moreover, the inhibitors of HMGA1, TRIP13 and c-Myc inhibited the stemness and EMT of QBC-939 cells with Wnt3a stimulation (Fig. 7b-d, Supplementary Figure 7E), and suppressed pCCA migration and invasion (Fig. 7e and f, Supplementary Figure 7F). All these results suggested that c-Myc promotes transcription and expression of *HMGA1* and *TRIP13*, and implicated that HMGA1-TRIP13 axis facilitates pCCA progression in a c-Myc-dependent positive feedback loop.

In our previous study, we showed that TCF family, important component of Wnt-β-catenin signaling, can induce c-Myc expression and promote pCCA progression [9], therefore we detected the correlation between HMGA1 and TCF family, which showed that HMGA1 regulated TCF4/TCF7/LEF1 expression and their downstream effector c-Myc (Supplementary Figure 9). This result suggests that HMGA1 has multiple crosslinks with Wnt-β-catenin-Myc signaling, which may be another positive feedback loop to amplify cell stemness and EMT in pCCA.

## Discussion

Identification of biomarkers for the selection of patients harboring pertinent genetic aberrations is an essential factor in targeted therapy. However, studies of biomarkers are lacking in pCCA compared with many other tumors due to the low radical pCCA resection rate and the difficulty of cohort establishment of patients with radical surgery. A molecular map is critical to direct targeted therapies and the future rational treatment of pCCA. In this study, mRNA sequencing of eight pairs of pCCAs and their adjacent normal bile duct tissues (the largest sample size of pCCA used for high-throughput sequencing to date) provided more detailed genetic landscape to pCCA and guide the exploration for effective biomarkers. Clinically, we identified the prognostic significance of HMGA1 and TRIP13 in pCCA. Interestingly, double positive expression of HMGA1 and TRIP13 was a more sensitive biomarker than HMGA1 or TRIP13 alone. Thus, postoperative detection of HMGA1 and TRIP13 could help stratify high-risk patients, guide individual treatments, even develop targeted therapies. In a previous study, Quintavalle reported that HMGA1 expression was up-regulated in CCA [20], but they did not classify different CCA histological type such as iCCA, pCCA or dCCA, which represented that iCCA may also have elevated HMGA1. High HMGA1 expression may be a common molecular feature of all CCA types irrespective of their anatomical origin, which needs further verification.

Histologically, differences in nuclear structures are the most important variations between cancer cells and normal cells. Chromatin binding proteins play key roles in maintaining nuclear organization, which is essential for expression of stem cell characteristics, both during development and tumorigenesis. HMG proteins, including 3 families: HMGB, HMGN, HMGA, all modify chromatin structure, although each family has distinct functions [13]. In our previous study, we demonstrated that HMGB1 promoted the recurrence and progression of pCCA via a paracrine pathway [35, 36]. Here, we showed that HMGA1 was a tumor promotor in pCCA and was correlated with progression and poor prognosis. Although *HMGA1* is known to be an oncogene, its tumor-promoting function is still not fully studied because its tissue-specificity and that it is involved in convergence of many signal pathways. Our findings indicated that HMGA1 promoted tumor progression via elevating *TRIP13* transcription and expression. As an architectural transcription factor, HMGA1 interacts with AT-rich regions in the minor groove of DNA via AT-hook domains, which mostly relies on the architectural structure of chromatin structure instead of special DNA sequences [37], so we did not try to map the interacting sequence of *TRIP13* promotor in the luciferase assay. Collectively, our data provided a new mechanism of HMGA1-induced progression of tumor and indicated a new treatment approach to CCA and other the HMGA1-overexpressing cancers.

**Fig. 8:**
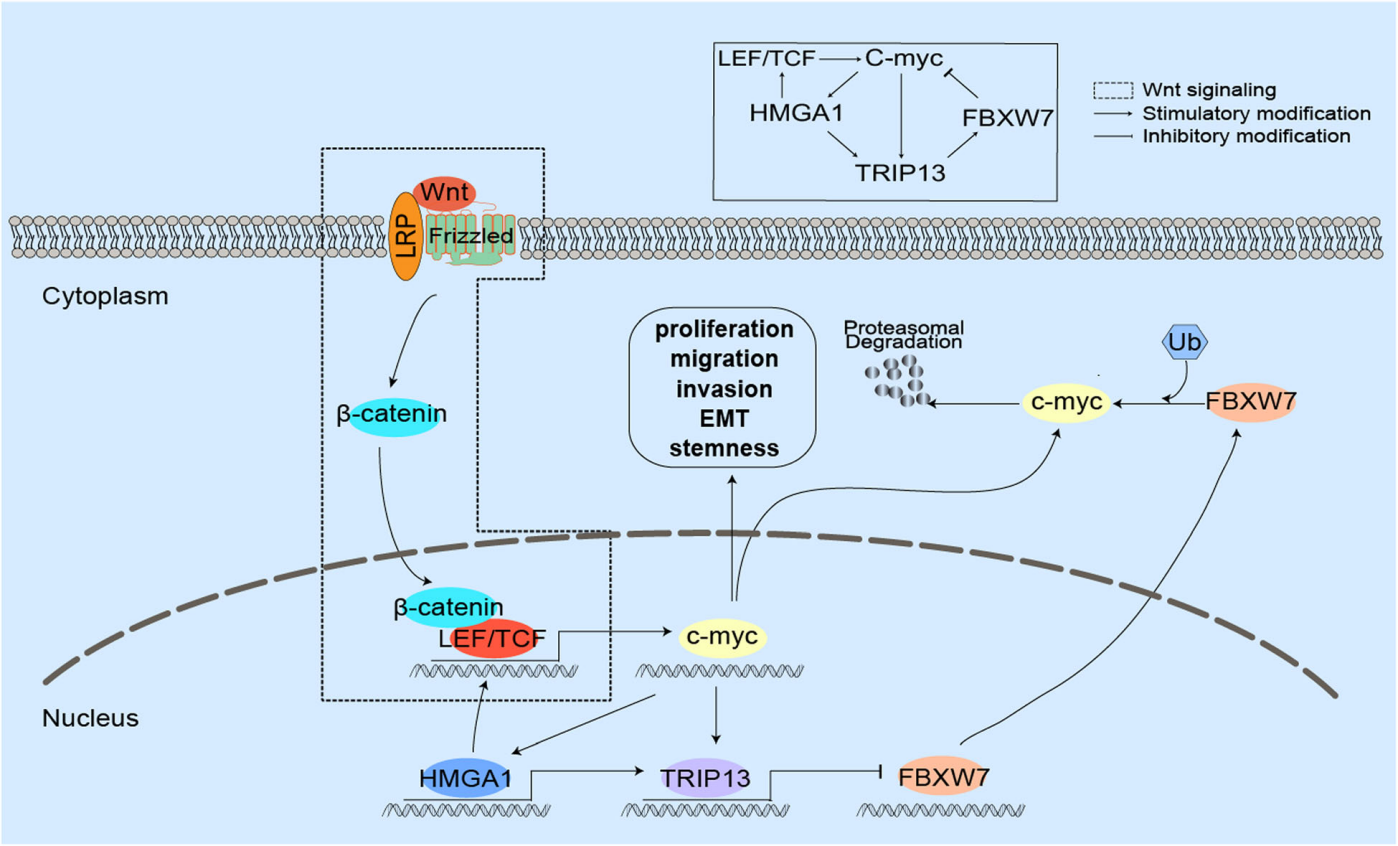
The schematic depiction of the positive feedback loop of HMGA1-TRIP13-c-Myc pathway. HMGA1 can induce the transcription and expression of *TRIP13*, therefore suppress FBXW7 expression and stabilize c-Myc, and eventually promote pCCA proliferation, migration, invasion, stemness and EMT. In the other side, c-Myc promoted *HMGA1* and *TRIP13* transcription, forming a positive feedback loop to amplify the effects of HMGA1-TRIP13 axis. Moreover, HMGA1 increased c-Myc expression via upregulating TCF family, which established another positive feedback pathway

HMGA1 is enriched in aggressive cancers and stem cells, and c-Myc is one of the four well-known Yamanaka factors influencing stemness [38]. In our study, HMGA1 can induce the transcription and expression of *TRIP13*, therefore suppress FBXW7 expression and stabilize c-Myc, and eventually promote pCCA proliferation, migration, invasion, stemness and EMT. In the other side, c-Myc promoted *HMGA1* and *TRIP13* transcription, forming a positive feedback loop to amplify the effects of HMGA1-TRIP13 axis. Moreover, HMGA1 increased c-Myc expression via upregulating TCF family, which established another positive feedback pathway (Fig. 8). This is the first report on the positive feedback loop between HMGA1-TRIP13 axis and c-Myc, providing the underlying mechanism of how HMGA1-TRIP13 axis promotes cell stemness and EMT. Moreover, we showed that TCF4 also influenced HMGA1 expression via elevating c-Myc. TCF4 and its target gene *c-Myc* are important nodes in Wnt-β-catenin signaling, which is an essential pathway affecting cell stemness and tumor progression. To date, the correlations between HMGA1 and Wnt signaling are controversial and have several important issues to solve. On one hand, HMGA1 amplified Wnt signaling and enhanced stemness by upregulating Wnt effectors with an elusive mechanism [13, 14]; on the other side, some evidence showing that Wnt-β-catenin/TCF signaling elevated HMGA1 expression [39, 40]. The paradox of HMGA1 and Wnt-β-catenin/TCF signaling got a new explanation in our study, which was that HMGA1 amplified Wnt signaling and stemness in a positive feedback pathway dependent on c-Myc involvement. This positive feedback effect of HMGA1-c-Myc nexus may not just suit pCCA, but also adjust other kinds of tumors, which could be a common molecular feature of tumor cells. If so, this would be a great breakthrough of HMGA1-induced tumor stemness. Breaking this HMGA1-TRIP13-c-Myc nexus would be a very promising approach to treat tumors because specific small-molecule inhibitors of them are available. The inhibitors of HMGA1, TRIP13 and c-Myc were all used to block the HMGA1-TRIP13-c-Myc nexus, and exhibited a CCA-suppressing effect. However, it is a long way from in vitro experiments to in vivo experiments. C-Myc inhibitor 10,058-F4 exhibited no significant anti-tumor activity because of its rapid metabolism and low concentration in tumors (Guo et al., 2009). Although DCZ0415 and netropsin had tumor-suppressing role in melanoma or medulloblastoma (Lau et al., 2012; Wang et al., 2020), their molecular structure should be modified continuously to improve the water-solubility and permeability to cell.

As a critical component of chromosome recombination and chromosome structure development during meiosis, the role of TRIP13 in stemness and EMT is rarely investigated. In this study, we showed that *TRIP13* transcription was promoted by HMGA1, and that TRIP13 promoted pCCA stemness and EMT. In our previous study, we proposed that FBXW7 suppressed stemness and EMT of CCA via mTOR signaling pathway [32]. Here we demonstrated that FBXW7 was responsible for the stemness and EMT induced by HMGA1-TRIP13 axis, and identified c-Myc as a new effector in this progress. Moreover, we demonstrated that TRIP13 was a prognostic biomarker of pCCA. All these results suggested the core function of TRIP13 in pCCA progression. The newly-developed small molecule inhibitor of TRIP13, DCZ0415, was applied in our study and exhibited significant effect to suppress pCCA progression. This suppressing role of DCZ0415 in pCCA implicated that it could be a promising target drug of pCCA.

## Conclusion

In summary, we for the first time identified HMGA1 and TRIP13 as prognostic biomarkers of pCCA. HMGA1 facilitated pCCA proliferation, migration, and invasion by promoting tumor stemness and the EMT, which required the involvement of TRIP13. Using in vitro and in vivo experiments, we demonstrated that TRIP13 promoted stemness and the EMT by suppressing *FBXW7* expression and stabilizing c-Myc. In turn, c-Myc can induce the transcription of *HMGA1* and *TRIP13* in pCCA cells. All data demonstrated that HMGA-TRIP13 axis promoted pCCA stemness and EMT in a positive feedback pathway dependent on c-Myc. Taken together, our findings suggested that postoperative detection of HMGA1 and TRIP13 could help stratify high-risk patients, thus guide individual treatments and facilitate the development of targeted therapies, and that breaking this HMGA1-TRIP13-c-Myc nexus may be a very promising approach to treat pCCA.

## Supplementary Information

**Additional file 1: Supplementary Figure 1.** HMGA1 expression in cell lines. **Supplementary Figure 2.** HMGA1 promoted pCCA progression. **Supplementary Figure 3.** TRIP13 promoted pCCA migration, invasion and stemness. **Supplementary Figure 4.** TRIP13 was required in HMGA1-induced pCCA progression. **Supplementary Figure 5.** FBXW7 suppressed TRIP13-induced progression. **Supplementary Figure 6.** HMGA1 and TRIP13 expression was correlated with FBXW7 in xenografts. **Supplementary Figure 7.** FBXW7 induced c-Myc degradation by promoting its ubiquitination. **Supplementary Figure 8.** HMGA1-TRIP13 axis promotes stemness and EMT in a positive feedback pathway dependent on c-Myc. **Supplementary Figure 9.** HMGA1 regulated the expression of TCF family. **Supplementary Table 1.** The Expression of HMGA1 and TRIP13 in primary cohort and validated cohort. **Supplementary Table 2.** Primers for qRT-PCR. **Supplementary Table 3.** The information of sh/siRNA sequences. **Supplementary Table 4.** The promoter region sequences. **Supplementary Table 5.** Proteomic HMGA1-linked signature genes and up-regulated genes in exome and transcriptome sequencing profiles. **Supplementary Table 6.** The prognostic significance of HMGA1/TRIP13 and clinicopathological factors in pCCA. **Supplementary Table 7.** Correlation between TRIP13 and clinicopathological factors.

## Data Availability

All the data generated or analyzed in this study are included in this published article and its Additional files.

## Abbreviations

CCA: Cholangiocarcinoma
iCCA: Intrahepatic cholangiocarcinoma
pCCA: Perihilar cholangiocarcinoma
dCCA: Distal cholangiocarcinoma
HMGA1: High mobility group A1
TRIP13: Thyroid hormone receptor interactor 13
FBXW7: F-box/WD repeat-containing protein 7
IHC: Immunohistochemistry
siRNA: Small interfering RNA
shRNA: Short hairpin RNA
ANOVA: Analysis of variance
MOI: Multiplicity of infection
ROC: Receiver Operating Characteristic
qRT-PCR: Quantitative real-time PCR
SDS-PAGE: Sodium dodecyl sulfate polyacrylamide gel electrophoresis
PVDF: Polyvinylidene flfluoride
EMT: Epithelial Mesenchymal Transition
CCK-8: Cell counting kit-8
FBS: Fetal bovine serum
FPKM: Fragments per Kilobase Million
GO: Gene Ontology
TWIST1: TWIST family BHLH transcription factor 1
MCC: Mitotic checkpoint complex
KIFC1: Kinesin family member C1
LRRC59: Leucine-rich repeat-containing protein 59
TCGA: The Cancer Genome Atlas
MAL2: Myelin and lymphocyte protein 2
