# Correction: HMGA1-TRIP13 axis promotes stemness and epithelial mesenchymal transition of perihilar cholangiocarcinoma in a positive feedback loop dependent on c-Myc

**DOI:** 10.1186/s13046-025-03526-0

**Published:** 2025-09-15

**Authors:** Zhipeng Li, Jialiang Liu, Tianli Chen, Rongqi Sun, Zengli Liu, Bo Qiu, Yunfei Xu, Zongli Zhang

**Affiliations:** 1 Department of General Surgery, Qilu Hospital, Cheeloo College of Medicine, Shandong University, 107 Wenhuaxi Road, Jinan, Shandong 250012 China; 2 Department of General Surgery, Shandong Second Provincial General Hospital, Shandong Provincial ENT Hospital, Jinan, China

**Correction: J Exp Clin Cancer Res 40, 86 (2021)**

**https://doi.org/10.1186/s13046-021-01890-1**

Following the publication of the original article [1], the authors noticed that incorrect images were used in Fig. 6, Supplementary Fig. 3, and Supplementary Fig. 8, specifically:

- Fig. 6E-incorrect band of GAPDH for QBC-939 cells
- Supplementary Fig. 3A-incorrect representative image for shTRIP13-1 in the 24 h group
- Supplementary Fig. 8F-incorrect representative migration image in the Netropsin + /Wnt3A + group for FRH-0201 cells

The correct figures are provided below:

**Incorrect Fig.** 6

**Fig. 6:**
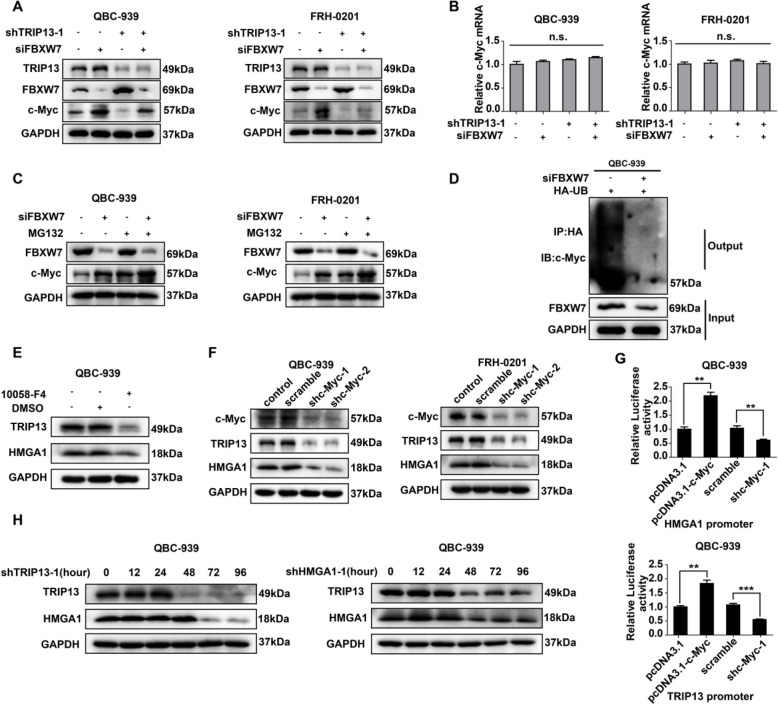
HMGA1-TRIP13 axis promotes invasion, stemness and EMT in a positive feedback pathway dependent on c-Myc. **a**, **b** WB (**a**) and qRT-PCR (**b**) showed that *FBXW7* and *TRIP13* knockdown regulated *c-Myc* expression but had little effect on *c-Myc* mRNA. **c** MG132 inhibited the FBXW7- induced *c-Myc* degradation. QBC-939(left) and FRH-0201(right) were incubated in 10 μM MG132 for 12 h before lysis. **d** *FBXW7* knockdown decreased the ubiquitination of c-Myc. 24 h after transfection with HA-Ub and siFBXW7, QBC-939 cells were incubated in 10 μM MG132 for 12 h. HA beads were used to precipitate HA-interacting proteins and c-Myc antibody was used to detect the ubiquitinated c-Myc. **e**, **f** c-Myc inhibitor 10,058-F4 (**e**) and *c-Myc* knockdown (**f**) decreased the expression of HMGA1 and TRIP13 in pCCA cells. 10,058-F4(10 μM) was used to pre-incubate QBC-939 cells for 12 h. g Luciferase assays revealed that c-Myc promoted the transcription of *TRIP13* and *HMGA1* of QBC-939 cells. The transcriptional activity of *HMGA1*(up) and *TRIP13*(bottom) were detected with luciferase assays. ** represents *P* < *0.01*, calculated with T-test. **h** HMGA1 knockdown rapidly decreased *TRIP13* expression, while *TRIP13* knockdown attenuated *HMGA1* expression 12–24 h later. QBC-939 were transfected with shTRIP13 or shHMGA1 and incubated for 0–96 h. Analyzed data were from three independent experiments, and each subgroup was performed at least in triplicate

**Correct Fig.** 6

**Fig. 6:**
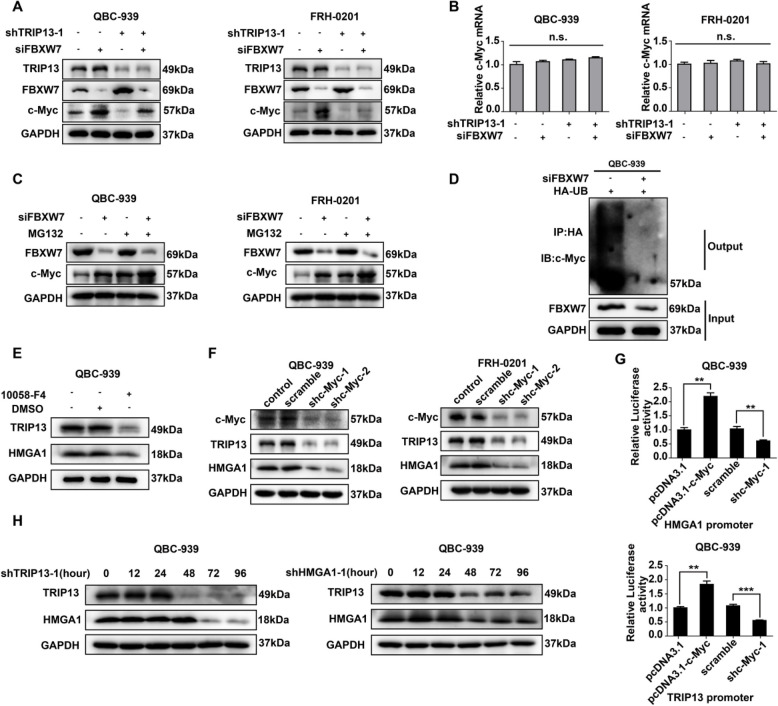
HMGA1-TRIP13 axis promotes invasion, stemness and EMT in a positive feedback pathway dependent on c-Myc. **a**,** b** WB(**a**) and qRT-PCR (**b**) showed that *FBXW7* and *TRIP13* knockdown regulated *c-Myc* expression but had little effect on *c-Myc* mRNA. **c** MG132 inhibited the FBXW7- induced *c-Myc* degradation. QBC-939(left) and FRH-0201(right) were incubated in 10 μM MG132 for 12 h before lysis. **d** *FBXW7* knockdown decreased the ubiquitination of c-Myc. 24 h after transfection with HA-Ub and siFBXW7, QBC-939 cells were incubated in 10 μM MG132 for 12 h. HA beads were used to precipitate HA-interacting proteins and c-Myc antibody was used to detect the ubiquitinated c-Myc. **e**,** f** c-Myc inhibitor 10,058-F4(**e**) and *c-Myc* knockdown (**f**) decreased the expression of HMGA1 and TRIP13 in pCCA cells. 10,058-F4(10 μM) was used to pre-incubate QBC-939 cells for 12 h. g Luciferase assays revealed that c-Myc promoted the transcription of *TRIP13* and *HMGA1* of QBC-939 cells. The transcriptional activity of *HMGA1*(up) and *TRIP13*(bottom) were detected with luciferase assays. ** represents *P* < *0.01*, calculated with T-test. **h** HMGA1 knockdown rapidly decreased *TRIP13* expression, while *TRIP13* knockdown attenuated *HMGA1* expression 12–24 h later. QBC-939 were transfected with shTRIP13 or shHMGA1 and incubated for 0–96 h. Analyzed data were from three independent experiments, and each subgroup was performed at least in triplicate

**Incorrect Supplementary Fig. 3:**


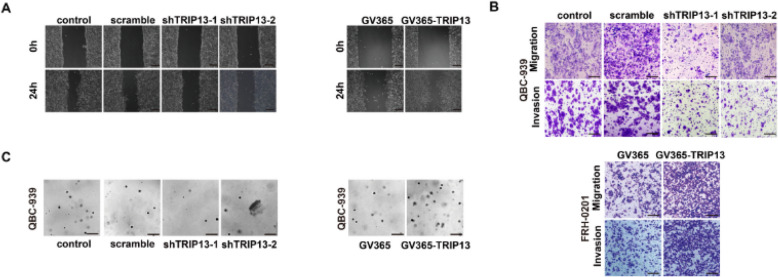


(A) Representative images of wound healing assay in Fig. 3F. Scale bar: 200 μm. (B) Representative images of transwell assay in Fig. 3G. Scale bar: 50 μm. (C) Representative images of 3D cell sphere assay in Fig. 3H. Scale bar: 200 μm

**Correct Supplementary Fig. 3:**


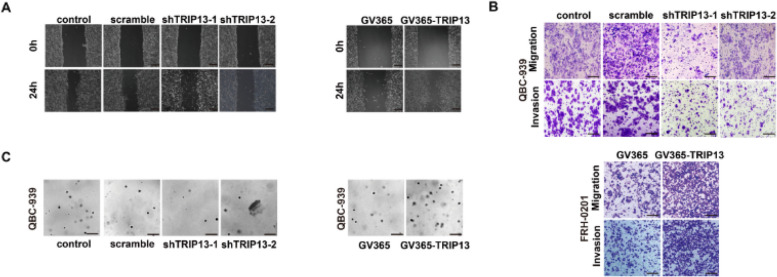


(A) Representative images of wound healing assay in Fig. 3F. Scale bar: 200 μm. (B) Representative images of transwell assay in Fig. 3G. Scale bar: 50 μm. (C) Representative images of 3D cell sphere assay in Fig. 3H. Scale bar: 200 μm

**Incorrect Supplementary Fig. 8**


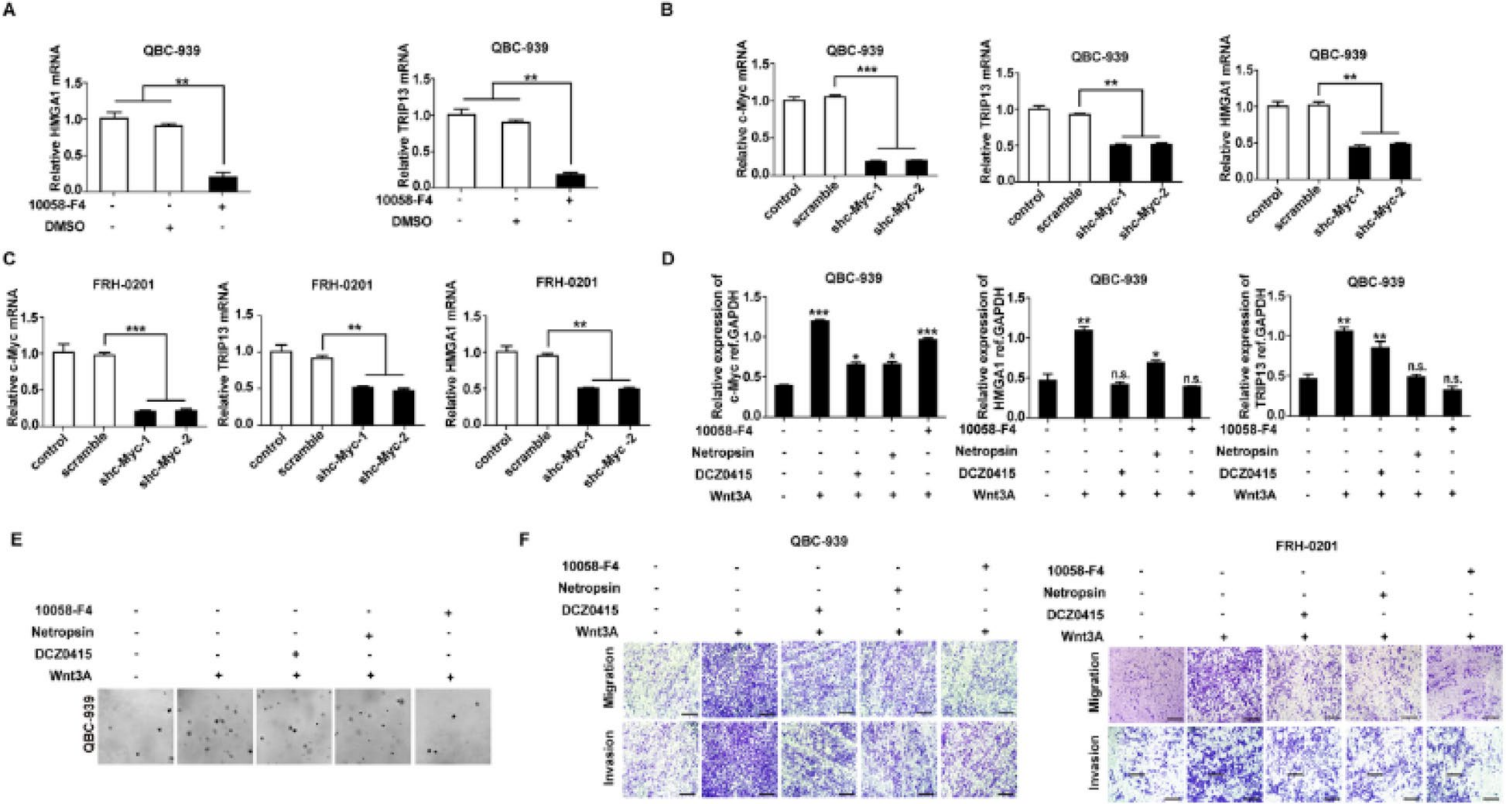


(A) In QBC939 cells treated with c-Myc inhibitor 10,058-F4(10 μM), qRT-PCR showed that 10,058-F4 decreased expression of *HMGA1* and *TRIP13* mRNA. (B and C)In QBC-939(B) and FRH-0201(C) cells, *c-Myc* was knocked down shMyc. qRT-PCR showed that *c-Myc* knockdown decreased mRNA level of HMGA1 and TRIP13. (D) Quantification of c-Myc, TRIP13 and HMGA1 in Fig. 7A. (E) Representative images of 3D cell sphere assay in Fig. 7C. (F)Representative images of transwell assay in Fig. 7E and 7F. Scale bar: 50 μm. * represents P < 0.05, ** represents P < 0.01, *** represents P < 0.001 compared with control or indicated groups, analyzed with T-tests

**Correct Supplementary Fig. 8**


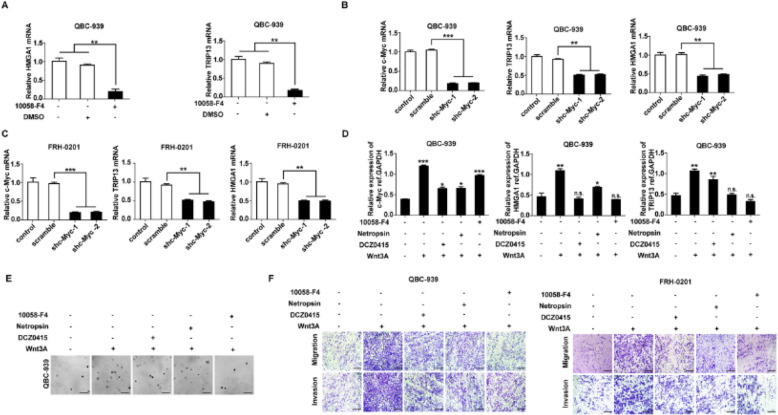


(A) In QBC939 cells treated with c-Myc inhibitor 10,058-F4(10 μM), qRT-PCR showed that 10,058-F4 decreased expression of *HMGA1* and *TRIP13* mRNA. (B and C)In QBC-939(B) and FRH-0201(C) cells, *c-Myc* was knocked down shMyc. qRT-PCR showed that *c-Myc* knockdown decreased mRNA level of HMGA1 and TRIP13. (D) Quantification of c-Myc, TRIP13 and HMGA1 in Fig. 7A. (E) Representative images of 3D cell sphere assay in Fig. 7C. Scale bar: 200 μm. (F)Representative images of transwell assay in Fig. 7E and 7F. Scale bar: 50 μm. * represents P < 0.05, ** represents P < 0.01, *** represents P < 0.001 compared with control or indicated groups, analyzed with T-tests

The corrections do not compromise the validity of the conclusions and the overall content of the article. The original article [1] has been updated.
