# Supplementary material for: HMGA1-TRIP13 axis promotes stemness and epithelial mesenchymal transition of perihilar cholangiocarcinoma in a positive feedback loop dependent on c-Myc
Source: J Exp Clin Cancer Res. 2021 Mar 1;40:86. doi: 10.1186/s13046-021-01890-1 (PMC7923631; doi:10.1186/s13046-021-01890-1)
Supplement: Supplementary file 1 — Additional file 1: Supplementary Figure 1. HMGA1 expression in cell lines. Supplementary Figure 2. HMGA1 promoted pCCA progression. Supplementary Figure 3. TRIP13 promoted pCCA migration, invasion and stemness. Supplementary Figure 4. TRIP13 was required in HMGA1-induced pCCA progression. Supplementary Figure 5. FBXW7 suppressed TRIP13-induced progression. Supplementary Figure 6. HMGA1 and TRIP13 expression was correlated with FBXW7 in xenografts. Supplementary Figure 7. FBXW7 induced c-Myc degradation by promoting its ubiquitination. Supplementary Figure 8. HMGA1-TRIP13 axis promotes stemness and EMT in a positive feedback pathway dependent on c-Myc. Supplementary Figure 9. HMGA1 regulated the expression of TCF family. Supplementary Table 1. The Expression of HMGA1 and TRIP13 in primary cohort and validated cohort. Supplementary Table 2. Primers for qRT-PCR. Supplementary Table 3. The information of sh/siRNA sequences. Supplementary Table 4. The promoter region sequences. Supplementary Table 5. Proteomic HMGA1-linked signature genes and up-regulated genes in exome and transcriptome sequencing profiles. Supplementary Table 6. The prognostic significance of HMGA1/TRIP13 and clinicopathological factors in pCCA. Supplementary Table 7. Correlation between TRIP13 and clinicopathological factors. [file 13046_2021_1890_MOESM1_ESM.docx]

**Supplementary Materials for**

HMGA1-TRIP13 axis promotes EMT and stemness of perihilar cholangiocarcinoma in a positive feedback loop dependent on c-Myc

Zhipeng Li MD et al.

**Supplementary Figures**

**Supplementary Figure 1. HMGA1 expression in cell lines.**


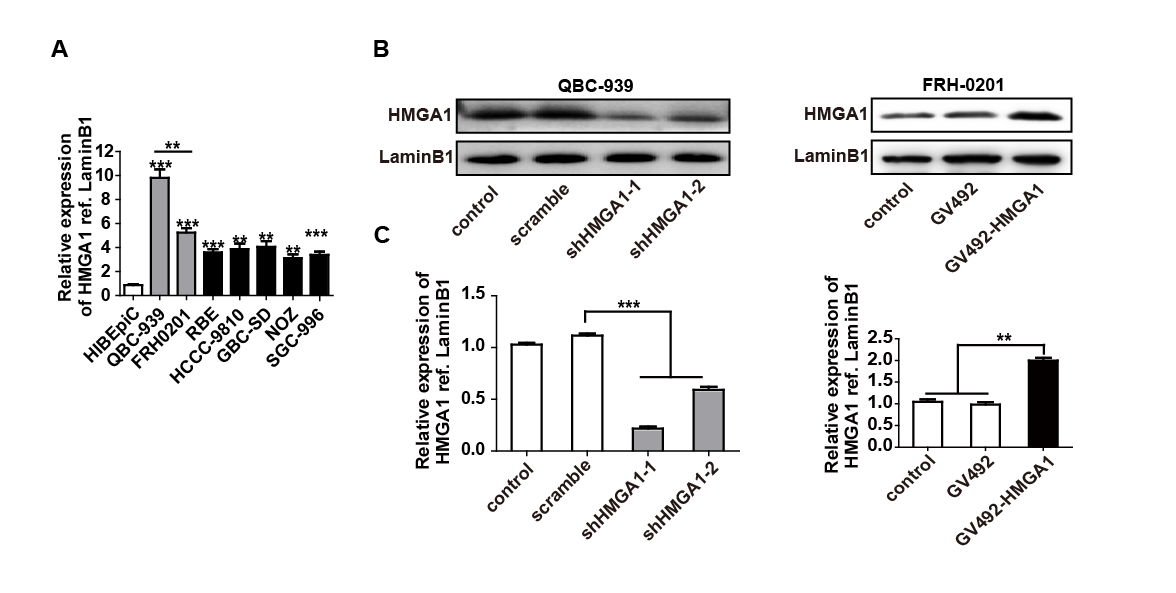


(A) Quantification of HMGA1 expression in Figure 2A. (B) HMGA1 expression was knocked down in QBC-939 and overexpressed in FRH-0201. (C) Quantification of HMGA1 expression in (B).

**Supplementary Figure 2. HMGA1 promoted pCCA progression**


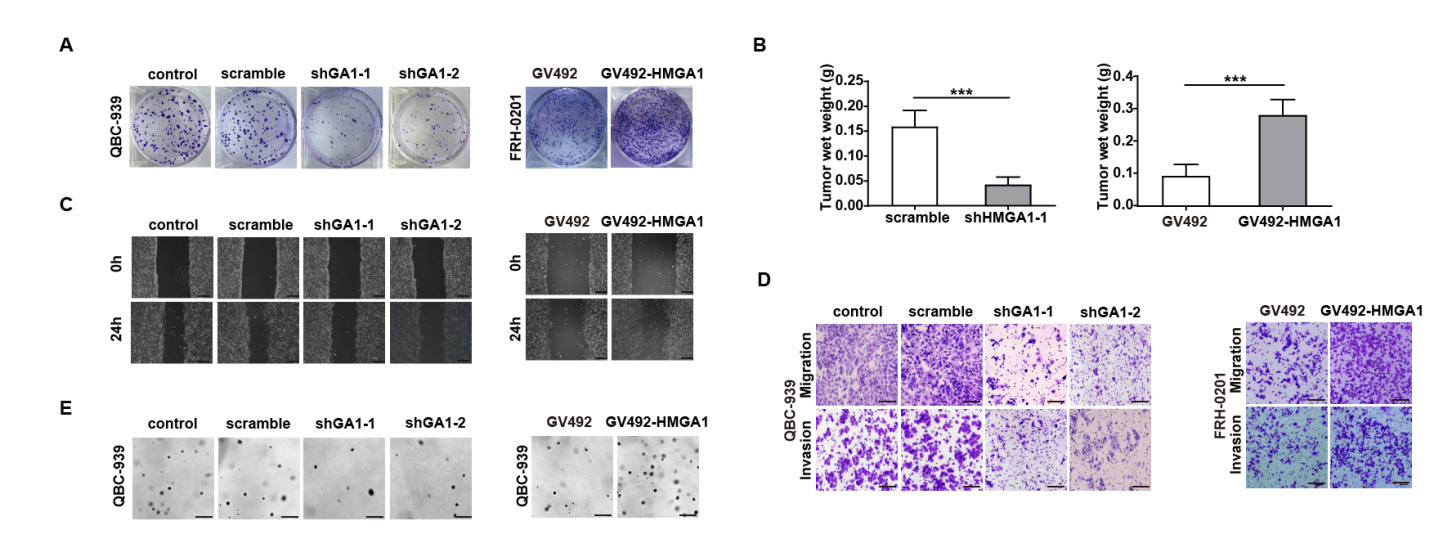


(A) Representative images of colony formation in Figure 1H. (B) Tumor weights of xenografts in Figure 1I were measured after 18 days (HMGA1 overexpression) or 21 days (HMGA1 knockdown). (C) Representative images of wound healing assay in Figure 1K. Scale bar: 200 μm. (D) Representative images of transwell assay in Figure 1L. Scale bar: 50 μm. (E) Representative images of 3D cell sphere assay in Figure 1M. Scale bar: 200 μm. ** represents P<0.01, *** represents P<0.001 compared with control or indicated groups, analyzed with T-tests.

**Supplementary Figure 3. TRIP13 promoted pCCA migration, invasion and stemness**


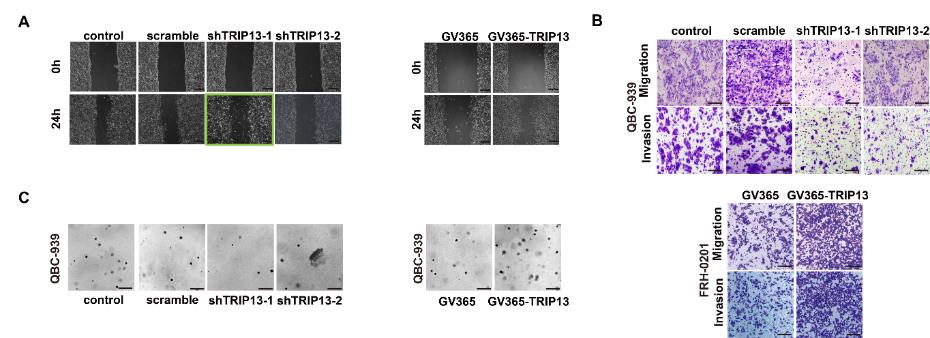


(A) Representative images of wound healing assay in Figure 3F. Scale bar: 200 μm. (B) Representative images of transwell assay in Figure 3G. Scale bar: 50 μm. (C) Representative images of 3D cell sphere assay in Figure 3H. Scale bar: 200 μm.

**Supplementary Figure 4. TRIP13 was required in HMGA1-induced pCCA progression**


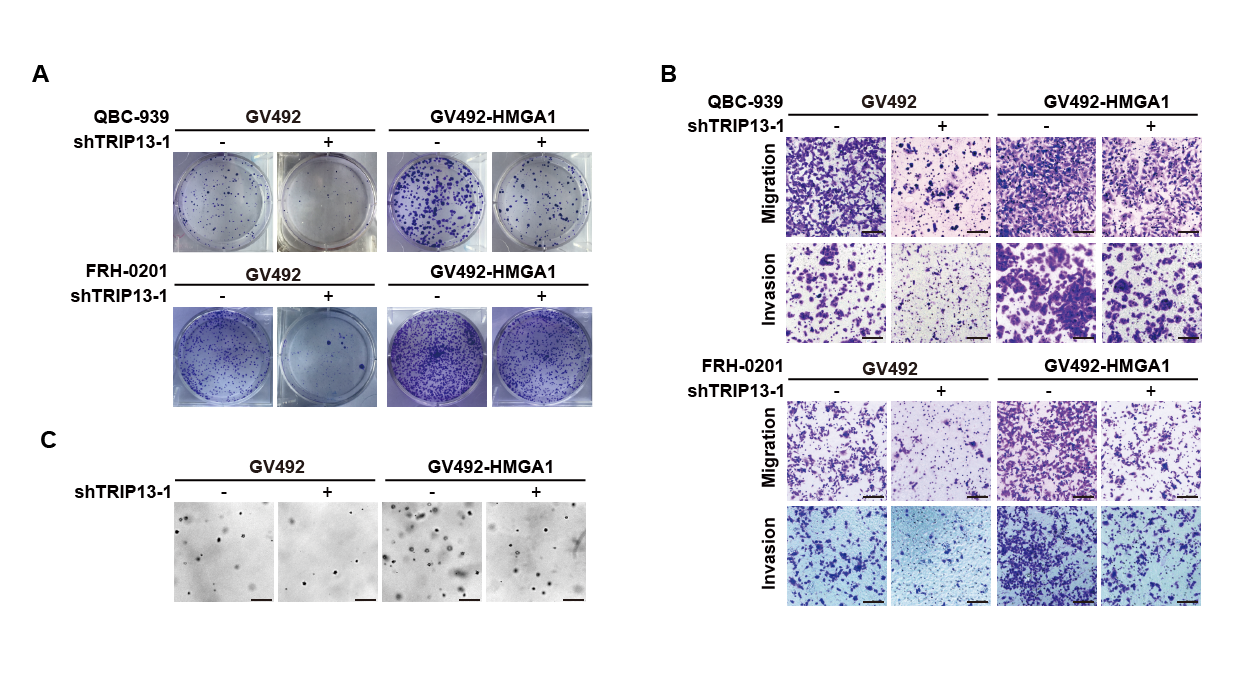


(A) Representative images of colony formation in Figure 4B. (B) Representative images of transwell assay in Figure 4E and 4F. Scale bar:50 μm. (C) Representative images of 3D cell sphere assay in Figure 4I. Scale bar: 200 μm.

**Supplementary Figure 5. FBXW7 suppressed TRIP13-induced progression**


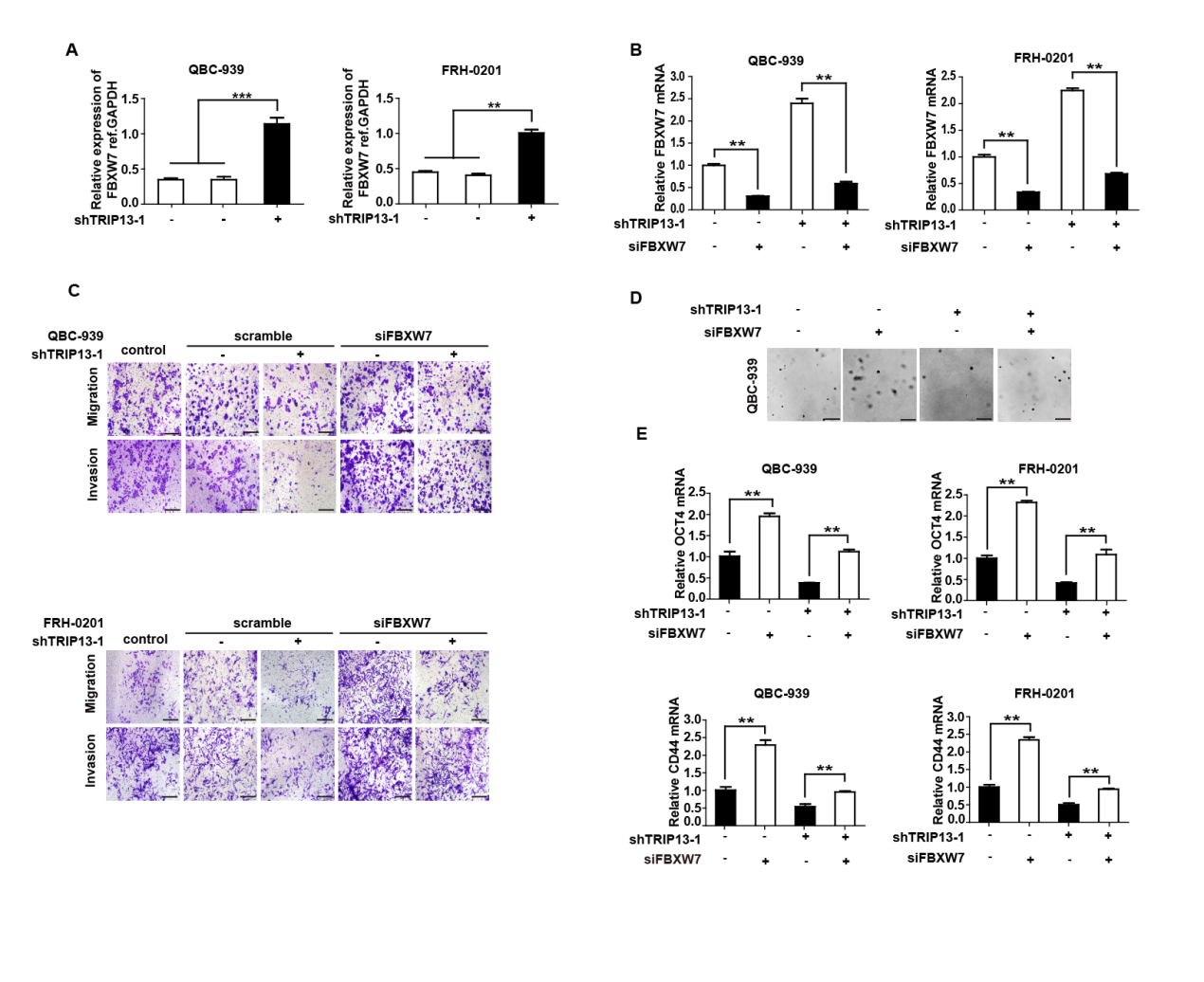


(A) Quantification of FBXW7 expression in Figure 5A. (B)qRT-PCR was used to detect the expression of FBXW7 in QBC-939 and FRH-0201 cells after TRIP13 knockdown. (C) Representative images of transwell assay in Figure 5D. Scale bar:50 μm. (D) Representative images of 3D cell sphere assay in Figure 5G. Scale bar: 200 μm. (E)In QBC-939 and FRH-0201 cells, qRTPCR was performed to detect the expression of cancer stem cell markers (CD44 and OCT4) after FBXW7 and/or TRIP13 knockdown. * represents P<0.05, ** represents P<0.01, *** represents P<0.001 compared with control or indicated groups, analyzed with T-tests.

**Supplementary Figure 6. HMGA1 and TRIP13 expression was correlated with FBXW7 in xenografts**

**
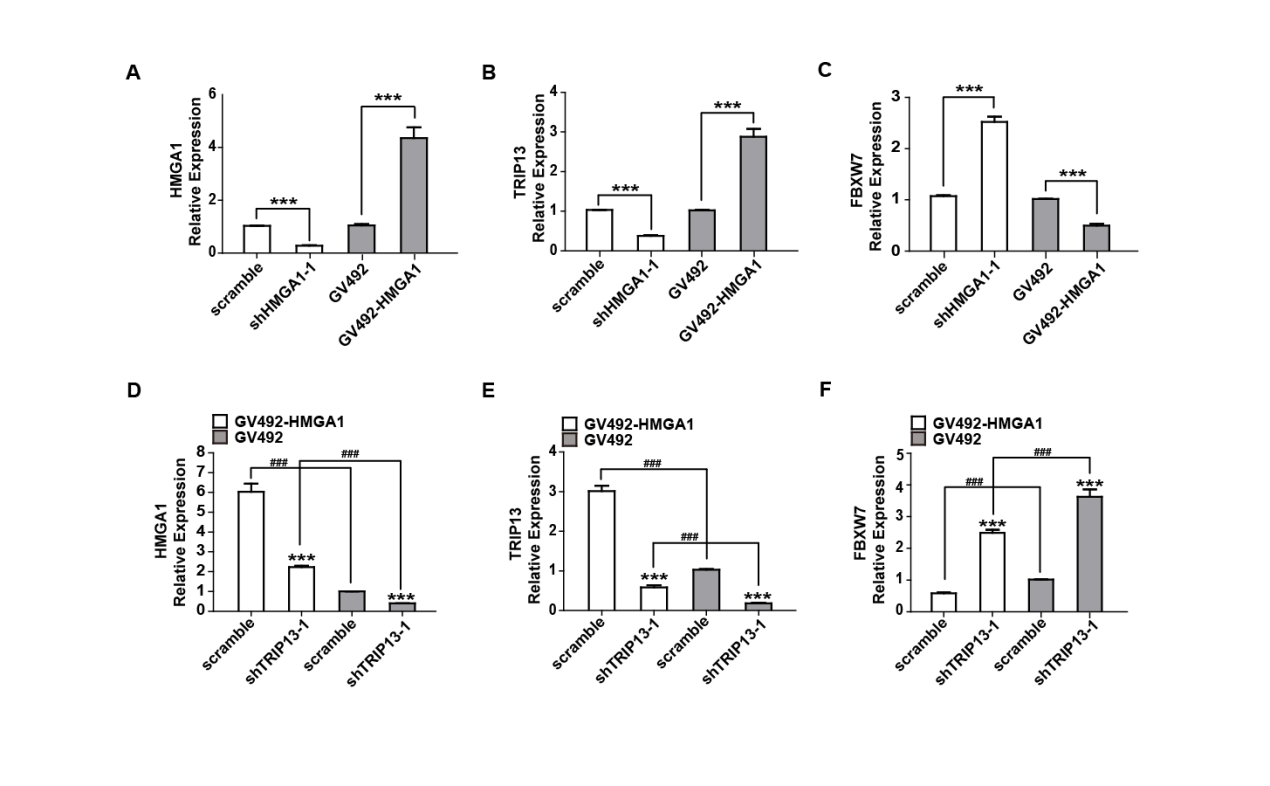
**

The relative mRNA level of *HMGA1*, *TRIP13*, and *FBXW7* were detected with qRT-PCR in the xenografts in Figure 1J (A-C)and Figure 4C(D-F). *TRIP13* mRNA was reduced when *HMGA1* was silenced, and elevated when *HMGA1* was overexpressed. On the contrary, *FBXW7* mRNA was elevated by *HMGA1* knockdown and decreased by *HMGA1* overexpression.

**Supplementary Figure 7. FBXW7 induced c-Myc degradation by promoting its ubiquitination.**


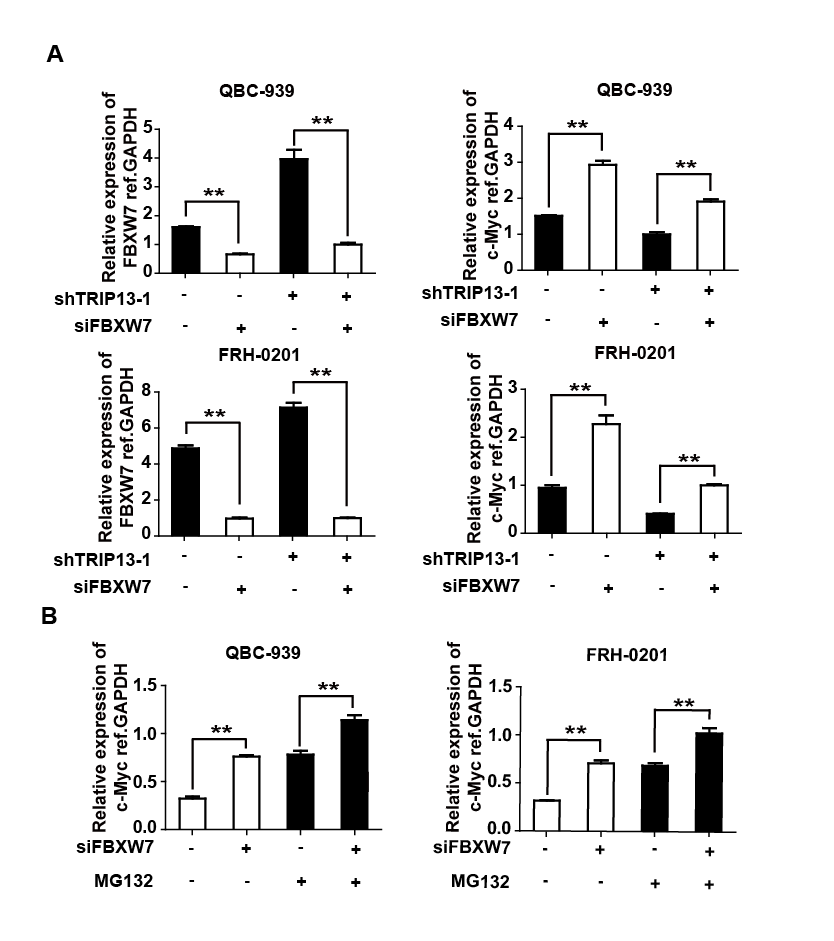


(A) Quantification of FBXW7 and c-Myc expression in Figure 6A. (B)Quantification of c-Myc expression in Figure 6C. ** represents P<0.01 compared with indicated groups, analyzed with T-tests.

**Supplementary Figure 8. HMGA1-TRIP13 axis promotes stemness and EMT in a positive feedback pathway dependent on c-Myc**


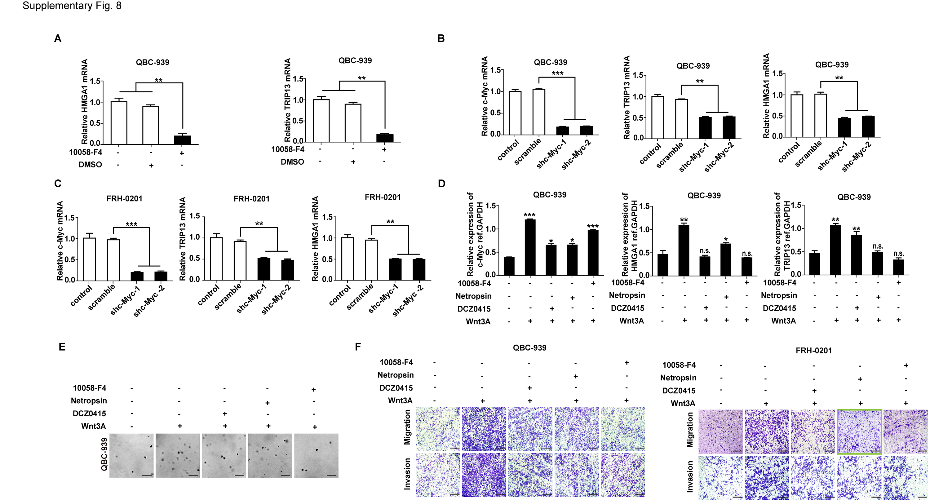


(A) In QBC939 cells treated with c-Myc inhibitor 10058-F4(10 μM), qRT-PCR showed that 10058-F4 decreased expression of *HMGA1* and *TRIP13* mRNA. (B and C)In QBC-939(B) and FRH-0201(C) cells, *c-Myc* was knocked down shMyc. qRT-PCR showed that *c-Myc* knockdown decreased mRNA level of HMGA1 and TRIP13. (D) Quantification of c-Myc, TRIP13 and HMGA1 in Figure 7A. (E) Representative images of 3D cell sphere assay in Figure 7C. (F)Representative images of transwell assay in Figure 7E and 7F. Scale bar: 50 μm. * represents P<0.05, ** represents P<0.01, *** represents P<0.001 compared with control or indicated groups, analyzed with T-tests.

**Supplementary Figure 9. HMGA1 regulated the expression of TCF family**


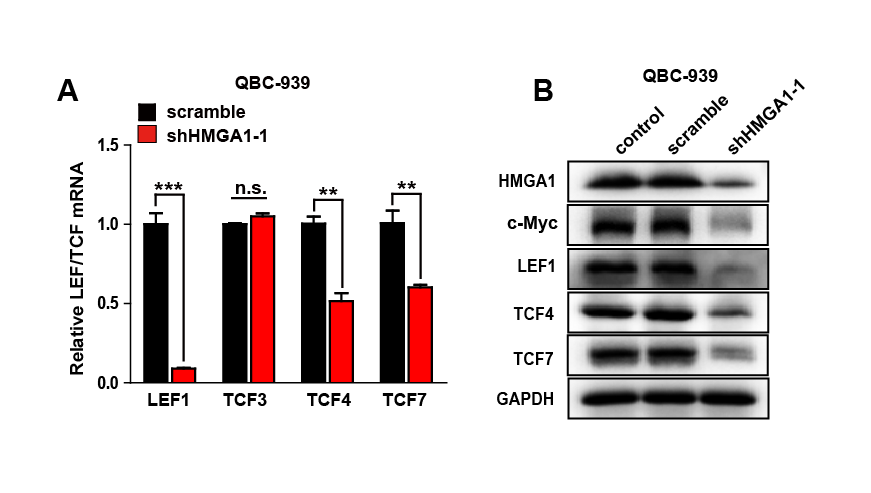


WB(A) and qPCR(B) showed that HMGA1 knockdown decreased expression of c-Myc and TCF family in QBC-939.

**Supplementary Tables**

**Supplementary Table 1. The Expression of HMGA1 and TRIP13 in primary cohort and validated cohort**

| **Characteristics** | Primary cohort n=325 | Validated cohort n=106 | Expression of HMGA1 | | | Expression of TRIP13 | | |
| --- | --- | --- | --- | --- | --- | --- | --- | --- |
|  |  |  | Low (n=35) | High (n=71) | | Low (n=32) | High (n=74) | |
| **Age（y）** |  |  |  | | |  | | |
| **<65** | 170 | 55 | 22 | | 33 | 19 | | 36 |
| **≥65** | 155 | 51 | 13 | | 38 | 13 | | 38 |
| **Gender** |  |  |  | |  |  | |  |
| **Male** | 203 | 76 | 26 | | 50 | 22 | | 54 |
| **Female** | 122 | 30 | 9 | | 21 | 10 | | 20 |
| **Tumor state** |  |  |  | |  |  | |  |
| **T1** | 108 | 35 | 15 | | 20 | 11 | | 24 |
| **T2/T3/T4** | 217 | 71 | 20 | | 51 | 21 | | 50 |
| **Lymphatic invasion** |  |  |  | |  |  | |  |
| **Negative** | 211 | 65 | 28 | | 37 | 24 | | 41 |
| **Positive** | 114 | 41 | 7 | | 34 | 8 | | 33 |
| **Distant metastasis** |  |  |  | |  |  | |  |
| **Absent（M0）** | 317 | 104 | 35 | | 69 | 31 | | 73 |
| **Present（M1）** | 8 | 2 | 2 | | 0 | 1 | | 1 |
| **Differentiation** |  |  |  | |  |  | |  |
| **Well/Moderately** | 198 | 63 | 17 | | 46 | 17 | | 46 |
| **Poorly** | 127 | 43 | 18 | | 25 | 15 | | 28 |
| **Tumor size** |  |  |  | |  |  | |  |
| **<3cm** | 218 | 58 | 22 | | 36 | 19 | | 39 |
| **≥3cm** | 107 | 48 | 13 | | 35 | 13 | | 35 |
| **TNM stages** |  |  |  | |  |  | |  |
| **I-II** | 199 | 62 | 28 | | 34 | 24 | | 38 |
| **III-IV** | 126 | 44 | 7 | | 37 | 8 | | 36 |

**Supplementary Table 2 Primers for qRT-PCR**

| Genes | Forward primer(5’-3’) | Reverse primer(5’-3’) |
| --- | --- | --- |
| HMGA1 | GGAAAAGGACGGCACTGAGA | TGGTGGTTTTCCGGGTCTTG |
| TRIP13 | TGCTGATTGATGAGGTGGAGAG | GGTTGCACAAGTATCACGCA |
| LRRC59 | TACCATGACCAAGGCCGGTA | GCCACAGAAATCCGACGGTA |
| KIFC1 | GGTGGTAGTGCTAAGATGCTCA | TTCACTTCCTGTTGGCCTGA |
| HDAC2 | CCATGGCGTACAGTCAAGGA | GCTTTATGGGGCCTATATATTTCCA |
| HSPD1 | TGCTCACCGTAAGCCTTTGG | TCTCCAAACACTGCACCACC |
| MSH2 | ACCAGCAGCAAAGAAGTGCT | TTGGAGTCGCACTGCCCT |
| RFC3 | AGTGCAACAATATCCTTTTACTGC | AACAGTTCTGAGAGAAGGCCC |
| SYNCRIP | GAGATGGTGCTGTCAAGGCTA | TTGGAGGGGGCATATGAGGT |
| SSB | GAACTTTGGGCCGCAGATG | TTGAAGTCGCCAAAATAATACTCAA |
| GAPDH | GAGTCAACGGATTTGGTCGT | GACAAGCTTCCCGTTCTCAG |
| OCT4 | TCAGGAGATATGCAAAGCAGAA | TTGCCTCTCACTCGGTTCTC |
| CD44 | CGTGGAGAAAAATGGTCGCT | TGTGGGCAAGGTGCTATTGAA |
| C-Myc | TACAACACCCGAGCAAGGAC | GAGGCTGCTGGTTTTCCACT |
| TCF7 | GTACAAAGAGACCGTCTACTCC | GGCTGTTGAAATGTTCGTAGAG |
| TCF4 | CTCCTGTGAGCAGTGGGAAA | CATAGTTCCTGGACGGGCTT |
| TCF3 | GCACTGGCCTCGATCTACTC | GTCGTAGCTGGGCGATAAGG |
| LLEF1 | CCCGTGAAGAGCAGGCTAAA | TCTGGACCTGTACCTGATGC |
| FBXW7 | CCACTGGGCTTGTACCATGT | GGTCCACTCCAGCTCTGAAA |

**Supplementary Table 3. The information of sh/siRNA sequences**

| **Name** | **The target sequence of shRNAs and siRNA** |
| --- | --- |
| shHMGA1-1(5’-3’) | CAACTCCAGGAAGGAAACCAA |
| shHMGA1-2(5’-3’) | GAAGTGCCAACACCTAAGAGA |
| shTRIP13-1(5’-3’) | GCTGGTAACCAAGATGTTT |
| shTRIP13-2(5’-3’) | GCAGTCTGTGTCTATTATTGA |
| sh-c-Myc-1(5’-3’) | CCTGAGACAGATCAGCAACAA |
| sh-c-Myc-2(5’-3’) | AAATTCGAGCTGCTGCCC |
| siFBXW7(5’-3’) | GCACTCTATGTGCTTTCA |
| **Name** | **The sequence of negative control shRNAs** |
| sh-scramble -HMGA1(5’-3’) | TTCTCCGAACGTGTCACGT |
| sh-scramble -TRIP13 (5’-3’) | TTCTCCGAACGTGTCACGT |
| sh-scramble-c-Myc(5’-3’) | TTCTCCGAACGTGTCACGT |
| scrambled FBXW7(5’-3’) | CUCCGAACGUGUCACGU |

**Supplementary Table 4. The promoter region sequences**

| Name | The sequences |
| --- | --- |
| HMGA1 promoter region sequence | GAAACCGAACGCCCTCCAGCCACCACCACTCACGGCCTACTAAGCGCGCGCTCCGGGCCCAGGGCACAAAGATGGAGGGAGCGGCGCGATCCACCCGCCCCACCCCCACCCACCAGACAAAGGGGCAGGCCGCCCCTCCCCCAGCCTTCTTCGCGGGCCCCGCCGCCCTCTGCTCCCCTCCCCTCGGGCCGCCCTTTGGTGACTTCCTTTCCCCTCCAAGGGCGGCCCCGGGACTTCTCAGGAACTGCGTTTCACCTGGACGTGGGGCGGGGAGCCGGCGCGCAGAGGGCGGCCTCCGGGCCCACCCCTAGGCCGAGCCGGCCCCAGGCCCGCGCCCTCCCCTTTGAACCCGCGCTCCCGGCCCCTGGCGCCTTCCCGCCCAGGCGCCCCCGCCCCCACCCGGTCAAGCACGTGCTGCCCGGGCCCCGAGCGCTTCCCGCCGCACGGGTGGGGGCTGGGCCCACCCCCCGCCCGCTCCCCACCCCCACCGGAAACATTCCTGCCACATTCCTGCAACTGCAAGGCCCAGCCCGCCGCTCCCCACCCCGCCTCCGCGCCGGGCCCAGGGCTTTCCTGCGTCCCCTCCACCGCCGGCTCGCCCCCTGAGGAGGGGGCTGGGCCAGGGCCTCGGCTGACCGGGGAGGAAGAAGGGGAGCAGAGAAAAACATGAGTCACAGCCGTGTGTCACTGGAGCGCATTTCAATTCCCTGCATCACAGGAGGTGTGGAAGGCCGCCTCGGGGACCGGGCGCGGGAGGTGCGCCCGAGAAGGCCCCGGGCCGGCCTGCAGGGCGCGCCGCTCCGCCTGCGCCCTTTCCTCCCCCACCGCCCTCCCCGCCATCTTCCCCTTTGGCTTCCTTCTCGCTCGGTGCAACAAGTCTTTGTTAAGCCGGGCGCCGGGCGGGCCAGGCGTGGCGGAGATGGCCTGCGTGCTCGGCCCCTGCCCTCAAAGCGCTTCCAGGCGACCTCTGGCCCACCTTTTATTTTTATTTACAGTCCCAACGGAAGGGCCCAGGTCCCCAAGTGGGCCTGCGTATCTCCAGAACACCATCTAAGTCACCTCAAGGTATGAAGCCTCCCTTGGGTGTACCTGCCAACGAGCCAATCGTTGGTTTCGCTGGAAGGCTCCACCTTGATCATGGCTCGCTGGTGGCCATTAATAAAACACTTTGGATTTCACAAGTTTCACGTTTGAATTTCACAAGACTTGTATCTCACCAATCAGCCACACGCGGGTTGACACTGAAAGGCACATGCTGACACCTGTCCGCCCAGGGGAAATCTACCATCTCTCTATTTTAGTTAGGGGGCGGCGTGTAGGCCGCGGAATTCTCTAACGAGCGCGTTTCTCTTCACCCCCTGGGCCCGCGTGGACCCCCGTCCACCCCCACACGCCCTGGGGGGGGGGCCGGGCCCACACGCCCTGGAAGCCCCTAGAGGTGACTCTCCCTGGGACCCCTGTACCAGGGAGGAAGGATACCGCCACCCGTCACCACCCCCCGCCAGAAGCTCCTTCGTGACTCCTCTGCGCGTGCCTTCCCACACCTCCTCCGTCCGGGACTGCGAGGAGTGGGCGGTCGACTCGAGTTCGCAGCCCAGGCCTCCCACACGCCCCTCCCTGCCGTCAGCACCCACCCGCGCGGCAGCGGCGGCGGCGGCTGGCGGGCGGCCGCCCTTTTAAATCCCCGGGCTCATTTGCATGGCCCCGCCCCCTGAGTGACACGGCTGGCGCGGGCGGGCCCGTCCCCCCTGCCCCTGGGTCGCTCTTTTTAAGCTCCCCTGAGCCGGTGCTGCGCTCCTCTAATTGGGACTCCGAGCCGGGGCTATTTCTGGCGCTGGCGCGGCTCCAAGAAGGCGTGAGTTCGCGGCCGCTCCGGTGGCTTCTTTTTTTTATATCTATAATTTAATTAAATTATTTATTTATTGAGGCCGCGCACGGGCCGTGCCCAGCTTCCTGCCCCTCGCCATCCTTC |
| TRIP13 promoter region sequence | CTGAAGGGCACCAGTGGTAACTGGCCACGCCTTGCTCTAGACATTCAGAGACACCGGAGGAAACCTCAGGCCAGAGGACACCTGGGATGAGGCCCTGGAAATACCAGCTCTCCTCCCTCCCATGCTTCTCCCCAAAGCAACAGGACACGGTGCCGACCCCTCATTTACAACGATGAGCTGTGAACACCAGGAGAGTCTCTAAAAGTGCACCCTTGCCTGGCTGTGTCCGGCACGCTCGGACTGGCCGATCTGGGGGCGGCTCCACCTCCACCTTCTTCCCAGGATCAAAGTCGTCAGCCTCTCCCTCCGTGTCACAGTGCTCCCTCTCTCGCTTCCGCTTCTTCTCTTCCTGGGCGGCAGAGTCAAGGGAGTGAGAAAGGCAGGAGTAGGCGGGATCCGGACAGGTCTGCCCAATCCCCTCGCAGTTCTCAGGGGAGGGACAGAACTAAGGGAAACCCCCTCCGAGATCCTCCTCATGCCAGGCTCCCTCCTCACAGAGACCCTGGCAGGGTCTGCTGAGGAACAGCACACCCAGGATCCTGGAACTGAGTCTGCGGGCCTCAGCGGTTCGCGTTTTGCTACCAACGGAACACCCCCTCCCCTCCTCCAGCCACGCAGGCGCACTCTGCCTGGGTCCTGGGACCAGGCTCCCCACGGGCTCCTCGTGGGCAGCGTCCGGGCCACCGCCGCTGCTCCTTTACCTTTCGCTTCCTTCTTTCCTCATCGTCCAGATGCTTCTCCTTCTCGGACTTCTTCTTCTTCTTCTTTTTCTTTTCTTTGTGCCTCTCTCGCTCATGGTCTGACCTGTCATCATAGTAACTGGAGTCGTGGCCGGATCCTGAGAGTTCAGTCACTTCACTTCCTCCGACCTTCAGGACTAGCTTTAGAGGCTTCTCCAGGGGCTTGTCGGCATAATCTGCACAGACACAGACCGAGTTCTACCCGCGTGCTCAGGTGCAAACGCCACGCAGCCAGGTGAGACGTGAAGGTGCTAAGAGGGAAAGGCCTCCCTAAGGAGTACAGCGGGGCTGCCAACCAGCAGGGAAAGACGGAAGGGAAGACCACAGTGGCGGCATGTCACTGCCTCCTTGTTCTCCCTTCCCCTCCGCAGGGAGCTTCAGGTCCCTTTCGAGCCACGGTGTCCTCACCAGAGGCCCTGTGGGATGTTGTTCACATTACTCGTTCAAGAAGATGGATTAAGAGGGACCTGGAACCCCGGAGCCCGATCTCCCTCCTAGAAACCCCCAGCACTTCGGTTCACCCTCGCCTGACTTCCTGCAGGCGCCCGTGCTGCCCGCTGACACCCATGGAGCTGCTGCGGGAGCAAGGGAGAGGGAGGGAAAGGCGGGAGAAAGGGCAGCCCAGCTTCTCCCTGAGCTCCACACAAAAGCCAGCACAGATGCTTCCCTAGTCTCCAGGACCGGGGTGAGTGGGCTTGCAGCCTCGAACCCAGAACCCTCTACCAGGAACGTAAGCGCCTACCCAGGACCCCTCACGTGTGCCCGGTTCCCTGCCCGAAATCCCAGGACCCCCTCCCGCGTGCCCAGAACCCCTCCCTCGTGGCCAGGACCTCGCCCGGTGCCCAGGACCCCCGTCCGCGTGCCCGGAACTCCTCCCCCGTGCCCGGGACCCCGCCCGCCGCGCGTCACAAAGCGCCGCCGCCTCACCCTCGTAGGACGAGCGCCACTCGGCCTTGTGCTTCTTGTGCTTCTTGCCCATGGCGGCGCCGGCGGCGGGCCCGAGGCGGGGGCTGGGAACAGCTGGCACCCGGTCGGACCTTGGCCGCCACCGCCCCCTGGCCCTGGCTGGCCGCCCGCGCTCGCTGCGCCGAGGTTGCCGAGCTCGCTGGGCCGCGCCGGAAACGGGGCGAGGCGGGGCCGCGGCAGGAAGTGGCCCTGCCGGGCCCGAGCGCTTCCGGGTCAGGAGGTGGTGCGCCTCGCGCGGCAGATTCGAAGCTAGGGCGGGGCCCGCGGGCTGAGGCAGCGGCTGTGGCGGCGACGCTGGGCGTGAGGTGGCGGCGGCCGCGCCCTGGTTGGGTCCCCACTGCTCTCGGGGGCGCC |
| FBXW7 promoter region sequence | AAGCATGTGTATACAGGTATCTGTTCAAGTCTCTGCTTTTAATTATTTAAGGTATATACTCAGAAGTGGAAATGCTGGATCATGTGCTGATTTTATGTTTAAATTGTTGAGGAACTGCCATACTATTTTCTACAGCAGCTACATTCATTCATTTCACATTGCCGCCAAGGATGCACAAGGGTTCTGATTTCCCCACATCCTAGCCCATGCTTGTTGTTTACTGTTTTGTTTTGTTTTAAAACCATCCTAATGGATGTGAAGTGGTAGCGCATTGTGGCTTTGATTTGCATTTCCCTAATTAGTGATGTTGAGCATCTTTTCATGTGCCTATTGACCATTTACATATCTTTGGAGAAATCTGTTTTTGTGTAGCATTGCTGCCAGTGATTTTGCATTTTTAACTACATTTTAAATTGTGGTATAAGAGAAAGGTCTACTAATAGGAATGGGACTATTTCTTGTGCATAGATTGCCTTCCCAGCAGCCCACTTTAATATTTAGCAATGCTAATCTTTCCAAAGTCAAAAAGAAATCACCCATTTCTAACTTTCTCCTTTCAAATCCTTTCTTGTCTCCGTCAAAATTCCTGCTATAAAACTCCATAAAAGCTTGGAAGTTAAAAGTTGGAAGGAGAGATAAGTGGAGCTGACTGGCTGTTGGAAGAAGAAAATAGAATGGAAAGAGAGAATAGTTGATTGAGCACTGTGAATGGATAGTTCACAATCATTATCTACTATCTGTTAGGAATATGGATTATAGTATAAAGATTAATTAACACTTGCACAAATGGTTAATTTAGGGAAATATTTGAGATAATGCTGGAAAGTTTAGTTTGGGGCCACTTTGAAGAGAGTCTTCATCTCCAGACTTTATCTTAGGCCGTGGAGAACCATTGAAGGTTCTTGAGGAGTAAACGGCACACTGCATTCCCTCTCTGTGTTTTAGGAAGATTTATCTGACCATACTGTGAAGGAGAAATTAGGCACTAGGGATTTGCAATGGACTGGCTAGTTACTCTCCCATTTTACTATAATTACTTCCCATCATAGATTACTTTATAAATAAATACACTTTTTACATGTCTTTAATCAGTTGGGTGACTGTTATGCTTTCAAGTATAATCAATAAGTTTTTCAGAATTAAAGATTGACAATTGAAAACTCTGGTACAAAGATTAATCTGAAGTGTGTATTAATAGCTATTAGAGAACCAGAGTATACAGTATTTAATGTCTAGTGAGTATGAGATAAAGAATGGTGTGTATGGAAAAGGGAAAGAGAGCACACACTTGAAAATAGTGCTCATTATAATATATCTAAGGTAGAACAAACATACATATGTGTGTGTGTTTTTTTTAAACCATACGTAAACACAGTTCAAAAAATTCAAAACAAAATTTGTAACAGAATTCAAAACATTCCTAAAATATTAATGCTGACATTTGGATAAGGAAGACTTAATAGATACTCAAGATACAATGTTTGTTTTTTTTATTTTATTTTTGTAAGTAAAAATTTACTTTCACTTTCTAGGAAACACTCTTAGATTAGTTAGTTGGAGAAATATTAAAAGGGACCTTACAGCACAGCCCTGCTTTGGTTGTGTTCACATTACTGACTTTGTATGAGAGGTTCACCAGCTGTTTGACTGGCAGGAGCAGGAGCTTTCACTTCAGTAGTTTATAATTCCTGGCTATTCTAGGATAGTTTGCACTTCACCAAGCCTCAGACAGGTCAGGACATTTGGTAGGGGAAGGTTGAAAGACAAAAGCAGCAGGCCTTGGGTTCTCAGCCTTTTAAAAACTATTATTAAATATATATTTTTAAAATTTAGTGGTTAGAGCTTTTAGTAATGTGCCTGTATTACATGTAGAGAGTATTCGTCAACCAAGAGGAGTTTTAAA |

**Supplementary Table 5. Proteomic HMGA1-linked signature genes and up-regulated genes in exome and transcriptome sequencing profiles**

| **Proteomic HMGA1-linked signature**  **(21)** | **RNA-seq Total up(180)** | | | | |
| --- | --- | --- | --- | --- | --- |
| *ATAD2* | *HMGA1* | *OTX1* | *MMP3* | *TPT1P10* | *HNRNPA1P21* |
| *BAZ1B* | *TRIP13* | *TSPAN1* | *SLC7A11* | *HOXC9* | *LINC00659* |
| *COPS2* | *ARNTL2* | *TNFSF18* | *AP1S3* | *FAM83H* | *AL591806.1* |
| *CSF-1* | *HOXC8* | *BCL2L14* | *FAM92B* | *MUC16* | *AL590666.2* |
| *DDX18* | *FOXP3* | *KIF18A* | *TMEM171* | *GPR19* | *AP000695.1* |
| *GFPT1* | *POLQ* | *COL10A1* | *SIM2* | *MACC1* | *AL445933.1* |
| *GNAI3* | *LAMC2* | *PARD6B* | *COX6B2* | *LEMD1* | *LINC00278* |
| *DLGAP5* | *CDH3* | *RBPJL* | *TLCD1* | *CFAP73* | *PCAT7* |
| *ILF2* | *MCM10* | *POF1B* | *NIPAL1* | *KRT16* | *HOXB-AS3* |
| *KIF11* | *COL17A1* | *PAX8* | *ESM1* | *NPSR1* | *AP000695.2* |
| *KIFC1* | *SLC12A3* | *PRKCG* | *CSF2* | *GJB3* | *AC073150.1* |
| *LRRC59* | *GPRC5A* | *KRT17* | *DCSTAMP* | *CLDN4* | *LINC02195* |
| *NCAPG* | *CYP2W1* | *APOC1* | *CDX2* | *PAX8-AS1* | *AL365226.2* |
| *PGRMC1* | *IGSF9* | *TNS4* | *SMCO2* | *CTSE* | *BX470102.1* |
| *PRPF4B* | *SLC4A11* | *RHPN2* | *MMP10* | *S100A2* | *CPHL1P* |
| *RPRD1A* | *FXYD3* | *ALDH3B2* | *KLK6* | *LAMB3* | *RPLP0P2* |
| *RRM2* | *CDC45* | *CHIT1* | *EVPL* | *AC090673.1* | *CRNDE* |
| *SMC2* | *DSP* | *SDS* | *FAM83B* | *HOXC6* | *HOXC-AS2* |
| *TOP2A* | *MMP11* | *EMX1* | *TM4SF20* | *HOXC4* | *LINC01843* |
| *TRIP13* | *CENPM* | *KCNK1* | *KLK7* | *RF00019* | *LINC02099* |
| *WHSC1* | *PNPLA3* | *SCEL* | *PCSK9* | *IQANK1* | *AC105219.3* |
|  | *PLEK2* | *ABHD17C* | *RASSF6* | *LINC02408* | *MGAM2* |
|  | *SALL4* | *TFAP2A* | *COL22A1* | *CAPN8* | *AL049836.1* |
|  | *NTSR1* | *CELSR3* | *HTRA4* | *IGFL2* | *AL121790.2* |
|  | *FERMT1* | *TMPRSS4* | *MUC17* | *AC021218.1* | *HOXB7* |
|  | *PRSS33* | *TRIM29* | *CST2* | *KRT6A* | *AC104024.2* |
|  | *CEMIP* | *NUSAP1* | *HOXB9* | *ONECUT3* | *AC012531.1* |
|  | *ESRP1* | *STRA6* | *SDR16C5* | *SERPINB5* | *AL049555.1* |
|  | *SQLE* | *TRIM54* | *KRT19* | *IGHG4* | *AC124319.1* |
|  | *KCNN4* | *ANXA3* | *KRT15* | *CEACAM18* | *AC006262.1* |
|  | *CEACAM5* | *B4GALNT3* | *MAB21L4* | *C7orf71* | *ESPNP* |
|  | *HOXA13* | *RHCG* | *CRYBG2* | *AC007255.1* | *AC036176.3* |
|  | *HOXB6* | *NECTIN4* | *AL033381.1* | *AC007683.1* | *AL365181.2* |
|  | *GPRC5D* | *MAP3K21* | *ARL14* | *AL451139.1* | *AL365181.3* |
|  | *RASAL1* | *NT5DC4* | *FAM216B* | *ATP5MC1P4* | *AFAP1-AS1* |
|  | *ECT2* | *DNAJC5B* | *CCR8* | *AL031283.1* | *AC103702.2* |

**Supplementary Table 6. The prognostic significance of HMGA1/TRIP13 and clinicopathological factors in pCCA**

| **Clinicopathologic factors** | Univariate analysis | | Multivariate analysis  (HMGA1) | | Multivariate analysis  (TRIP13) | |
| --- | --- | --- | --- | --- | --- | --- |
|  | 3-year OS | P* | HR | P^#^ | HR | P^#^ |
| **Age (years)** |  |  |  |  |  |  |
| <65 | 0.551 | 0.012 | 1 |  | 1 |  |
| ≥65 | 0.506 |  | 1.758 | 0.046 | 1.597 | 0.098 |
| **Gender** |  |  |  |  |  |  |
| male | 0.674 | 0.341 |  |  |  |  |
| female | 0.148 |  |  |  |  |  |
| **Tumor size** |  |  |  |  |  |  |
| <3cm | 0.532 | 0.340 |  |  |  |  |
| ≥3cm | 0.459 |  |  |  |  |  |
| **Differentiation** |  |  |  |  |  |  |
| Well/Moderate | 0.566 | 0.240 |  |  |  |  |
| Poor | 0.302 |  |  |  |  |  |
| **T stage** |  |  |  |  |  |  |
| T1 | 0.520 | 0.041 | 1 |  | 1 |  |
| >T1 | 0.596 |  | 1.598 | 0.157 | 1.664 | 0.126 |
| **N stage** |  |  |  |  |  |  |
| N0 | 0.681 | <0.001 | 1 |  | 1 |  |
| N1/N2 | 0.259 |  | 2.802 | <0.001 | 3.111 | <0.001 |
| **M stage** |  |  |  |  |  |  |
| M0 | 0.764 | <0.001 | 1 |  | 1 |  |
| M1 | 0.000 |  | 11.654 | 0.002 | 14.567 | 0.001 |
| **TNM stage** |  |  |  |  |  |  |
| I＋II | 0.671 | <0.001 |  |  |  |  |
| III＋IV | 0.273 |  |  |  |  |  |
| **HMGA1** |  |  |  |  |  |  |
| Low | 0.305 | 0.004 | 1 |  |  |  |
| High | 0.591 |  | 1.874 | 0.072 |  |  |
| **TRIP13** |  |  |  |  |  |  |
| Low | 0.289 | 0.019 |  |  | 1 |  |
| High | 0.609 |  |  |  | 1.952 | 0.046 |

Abbreviations:OS=overall survival; HR=hazard ratio; 95%CI=95% confidence interval; pCCA=perihilar cholangiocarcinoma; * Calculated by log-rank test; ^#^ Calculated by Cox-regression Hazard model.

**Supplementary Table 7. Correlation between TRIP13 and clinicopathological factors.**

| **Characteristics** | **N** | **TRIP13**  **Low High** | | **P-value** |
| --- | --- | --- | --- | --- |
| **Age（years）** |  |  |  | 0.310 |
| **<65** | 55 | 19 | 36 |  |
| **≥65** | 51 | 13 | 38 |  |
| **Gender** |  |  |  | 0.734 |
| **Male** | 76 | 22 | 54 |  |
| **Female** | 30 | 10 | 20 |  |
| **Differentiation** |  |  |  | 0.384 |
| **Well/Moderately** | 63 | 17 | 46 |  |
| **Poorly** | 43 | 15 | 28 |  |
| **Tumor size (cm)** |  |  |  | 0.526 |
| **<3** | 58 | 19 | 39 |  |
| **≥3** | 48 | 13 | 35 |  |
| **T Stage** |  |  |  | 0.845 |
| **T1** | 35 | 11 | 24 |  |
| **T2/T3/T4** | 71 | 21 | 50 |  |
| **Lymphatic invasion** |  |  |  | 0.057 |
| **Negative** | 65 | 24 | 41 |  |
| **Positive** | 41 | 8 | 33 |  |
| **TNM stage** |  |  |  | 0.023 |
| **I+ II** | 62 | 24 | 38 |  |
| **III+ IV** | 44 | 8 | 36 |  |
